# Practitioner preferences in the analysis of cremation deposits in archaeology and biological anthropology: An overview of current osteoarchaeological practices with a focus on sex estimation

**DOI:** 10.1371/journal.pone.0310380

**Published:** 2024-12-02

**Authors:** Marta Hlad, Tessi Löffelmann, Jacob I. Griffith, Hannah F. James, Martine Vercauteren, Christophe Snoeck, Barbara Veselka

**Affiliations:** 1 Archaeology, Environmental Changes, and Geo-Chemistry (AMGC), Vrije Universiteit Brussel, Brussels, Belgium; 2 Research Unit Anthropology and Human Genetics, Faculty of Science, Université Libre de Bruxelles, Brussels, Belgium; 3 Department of Archaeology, Durham University, Durham, United Kingdom; University of the Witwatersrand, SOUTH AFRICA

## Abstract

Osteological data, such as biological sex, constitute a base for research in paleodemography and palaeopathology, as well as for understanding past socio-cultural practices. Despite extensive research efforts concerning cremated human remains over the past decades, an internationally acknowledged, standardized osteological protocol is not fully agreed upon. Furthermore, assessing cremation research practices from the literature is challenging because analysis reports are often written in the national languages of practitioners, which makes them difficult to access by an international audience. This study addresses this gap by directly reaching out to experts working with cremated human remains through an online questionnaire in Lime Survey. The aim is to identify and characterize patterns in cremation analysis practices. A particular emphasis was put on the methods of biological sex estimation. While the significance of these data is widely acknowledged, the ways in which they are obtained in practice are seldom examined. The results of this survey reveal an absence of standardization in protocols of analysis, and data collection, as well as in reporting on the cremated remains in publications and reports. Notably, the findings reveal regional preferences in methodological choices and inconsistent institutional training. A majority of respondents expressed a preference for traditional morphological methods over recently published metric and alternative methods for sex estimation. These variations underscore the complexity of establishing standardized cremation analysis protocols and highlight the importance of considering regional contexts and training in shaping future research practices.

## Introduction

Burnt human remains are encountered in many forensic and archaeological contexts. While all these remains underwent burning, the use of the term ‘cremation’ implies that the body was burnt in the course/context of a funerary rite [1]. The earliest known cremation funerary practices have been dated to the Mesolithic, but the rite has been practiced in a variety of ways across all continents in the past and is still used today [2–6]. The analysis of burnt human remains also has a long history of research within archaeology and forensic science [7]. Since the 1960s, researchers have tried to maximize data extraction from this highly fragmented and distorted material, transformed by fire.

A plethora of manuals and published recommendations exist regarding the osteoarcheological work with cremated remains [8–14]. With varying degrees of detail, some feature data recording forms [9, 10, 12] while others outline the protocol for analysis descriptively [14]. Thanks to the literature review by Gonçalves and Pires [15], different traditions, or ‘schools’, were identified as existing in France, the UK, Spain, and possibly Germany. However, no universal preference for one practice or the other seems to exist, which largely inhibits the comparability of the data produced. However useful literature reviews may be for grasping the state-of-the-art or synthesis of certain topics, they mostly assess articles that have been published in academic journals. Specifically in archaeology, grey literature is a major source of information [16]. This is the case because a large quantity of excavation work is produced commercially by private companies and conveyed in the form of site reports. Therefore, the accessibility of these data is variable, and the processing, at least in part, occurs in circles outside academia, if at all [17–19]. Due to these issues, a survey reaching out directly to the practitioners of these reports might allow new insights into current practices of analyses of cremation deposits.

Gonçalves and Pires [15] provide insight into how cremation deposits are assessed by authors from different countries. They found that while analytical goals tended to be similar, the influence of regional training traditions was visible in the literature. Age-at-death, sex, and the minimum number of individuals (MNI) were assessed by most authors, as was the color of the bones, which was used as a proxy for the maximum temperature reached during burning [20]. The representation of skeletal regions (e.g. whether the remains were collected from the pyre in an anatomical order) was recorded more rarely, as were stature and ancestry. The order of deposition of the remains into urns and deposits was limited to French and Italian scholars [15] (although personal communication by B.V. (Netherlands) and Elisavet Stamataki (Greece), indicated that other countries apply the same approach, even if this was not evident in the responses). In terms of the sex assessment, most of the examined papers (61.3%) used morphological methods rather than metric ones (28.8%), and 23.8% used a combination of these methods [15].

Numerous sex estimation methods are available to researchers and practitioners who work in the fields of archaeology, biological anthropology, and forensic science. Some of these methods are morphological (based on observations of sexually dimorphic features of the skeleton), while others are metric (based on measurements of dimorphic features). The most common areas of the skeleton used for morphological sex estimation are the pelvic bones and the bones of the skull [21–23]. Metric sex estimation methods exist for almost every bone and articular surface in the skeleton. Researchers have examined sexual dimorphism in measurements of long bones [24, 25], carpals and tarsals [26, 27], pelvis [23], crania [28], patellae [29], and even the hyoid bones [30], to cite just a few. Various schools and training traditions have different approaches to sex estimation preferring certain methods over others [31]. These disparities and the lack of standardization is known in the discipline, and concerns about the differences in practice and comparability of the results have been raised [15, 31, 32].

Cremation practices and burning in forensic settings complicate the matter further. Relatively little skeletal material is left after the body has been exposed to extreme temperatures. The fire consumes the soft tissues and organic components of the bones and turns them into fragmented and deformed calcined material [33] with varying degrees of shrinkage [11, 34–36]. As a result of burning and external factors such as fuel type, pyre management, funerary practices, comingling, and post-depositional processes [6], there is a significant variability in preservation and fragmentation in cremation deposits. In addition to this, excavation practices, handling, analysis approaches, and storage conditions can also influence the preservation of the remains. Moreover, since the organic matter is destroyed by high temperatures, DNA or proteomic analyses cannot be performed on this type of remains [10]. These factors constitute a major challenge for practitioners striving to estimate the sex of cremated individuals.

A series of methods for sexing cremation deposits and a small number of validation studies were published in the last 20 years [37–46]. Despite this research, practitioners working with cremation deposits mostly use “classical” morphological methods such as those outlined in Buikstra and Ubelaker [21] or Workshop of European Archaeologists [15, 22]. Practices and protocols for cremation analysis and specifically sex estimation of cremated remains between different groups of archaeologists and biological anthropologists can vary significantly. Two surveys have recently been conducted on practitioner preferences for the estimation of biological sex [31] and age-at-death [32] in unburnt skeletons. These surveys examined the methodological choices of practitioners in biological anthropology and forensic science. They sought to assess the consistency and standardization among professionals in these disciplines. The participants (n = 154) were mostly North American biological anthropologists and forensic scientists, although several participants from Europe and other continents also filled out the surveys. Both studies confirmed that practitioner choices for age-at-death and sex estimations are variable among different experts. The most concerning element of their reports was that several respondents reported using unpublished reference collections and methods available to them. Additionally, participants indicated that the level of experience of the practitioners played an important role in the quality of the generated data [31, 32].

This study aimed to obtain information on practices related to data collection, methods, and the reporting of the findings from cremation deposits from practitioners with different occupational backgrounds (commercial, public, and heritage sectors, academia). This survey was the first one conducted specifically for cremated human remains. The aim was to complement the information available from previous work concerning protocol standardization and reporting. Additionally, an attempt was made to establish relationships between the profiles of researchers and their practices related to the study of cremated remains. Special emphasis was put on methodologies of sex estimation, to assess the methods used, the perceived importance of sex data for archaeological research, and associated challenges (confidence in their estimates and the pressures they are facing) for the practitioners working with burnt human remains.

## Materials and methods

### Questionnaire

An online questionnaire was designed using Lime Survey (S1 File), containing 50 questions. The questions were divided into seven groups:

1. The participants
2. Level of training in the analysis of cremated remains
3. Working environment
4. Protocols used for cremation analysis
5. Questions on sex assessment
6. How results are reported
7. Comments and thoughts on estimating sex in cremation deposits.

The questionnaire was initially compiled in English and was translated into German and French before dissemination. The translation to German was done by T.L., and the translation to French by M.H., with respectively native and native-like level of these languages. All the questions were optional so that participants could continue to fill in the survey even if there were some questions they did not wish to or could not answer. The set of methodological questions was partially based on the questionnaire by Klales [31] so that results would be comparable to a certain extent. A set of questions also addressed the participants’ confidence when estimating sex in cremation deposits, and their thoughts on the importance and relevance of sexing cremated remains in archaeology.

The questionnaire was approved by the Ethics committee, as well as the data protection office of the Vrije Universiteit Brussel. It took approximately 30 minutes to complete and was disseminated through the authors’ professional network via emails and mailing lists (i.e. Jiscmail) and appropriate groups on social media (Facebook, X). Additionally, the announcement was distributed via the newsletter of the Société Préhistorique Française, and promoted to the participants of the session on biological profiling of cremated remains at the European Archaeology Association annual meeting 2023 in Belfast (session 401). The answers were collected between 10 of July 2023 and 31^st^ of October 2023.

### Participants

The inclusion criteria required participants to be professionals working with cremated remains in the field of archaeology or biological anthropology. This criterion was verified via combination of two questions: whether they are currently working with cremated remains, and how many cremated deposits they analyzed in their careers. In total, 56 questionnaires were collected of which 32 were complete submissions, and 24 were partial. Whenever results are reported here, the total number of answers is therefore noted. The percentages have been rounded to the closest integer.

### Data analysis

First, the data table exported from Lime Survey was ‘cleaned’ of answers that did not contain any data and of duplicate submissions. These were identified if two lines in the data table were identical in all respects. The translation of answers from French (by M.H.) and German (by T.L.) surveys into English was also undertaken. Quantitative data were analyzed in Excel, SPSS, and Python. Box- and strip plots were produced in Python’s Seaborn [47] and Matplotlib [48] packages. Scale data from the survey were plotted using the *plot-likert* package (https://pypi.org/project/plot-likert/). Statistical differences between any two groups were analyzed using Mann-Whitney U tests (none of the continuous variables were normally distributed, see S2 File). When multiple groups were compared, Kruskal-Wallis tests were used. For ordinal data, Wilcoxon signed rank tests were used, and the correlations were evaluated via Spearman’s rho. Whenever two categorical variables where compared, the Chi-square was calculated. For related samples (scale data), Friedman’s tests were performed. All the statistical tests were performed in IBM SPSS Statistics [49], and statistical significance was set at *p* < 0.05. Answers to open-ended questions were summarized and manually aggregated into different topics where appropriate. The process consisted of identifying topics evoked by the participants and grouping them based on recurrent keywords that emerged.

### Ethics

All the participants gave their informed consent before participating in this survey, by clicking on the tick box at the introduction site of the survey, where the purposes of the study and of the data collection were listed, along with the types of collected data. Only M.H. had access to the raw data and anonymized them as soon as they were retrieved. M.H. was also the only person analyzing the data to maximize the anonymity of the participants, given that the studied research community is very small. The only exceptions were the qualitative answers of German speaking participants which were translated by T.L. This study has received clearance from the Human Sciences Ethics Committee of the Vrije Universiteit Brussel, under the number ECHW_442.

## Results

Due to the high number of questions included in the questionnaire, many results were produced in this study, and it is not possible to include all of them in the main text of this article. Summary and descriptive statistics for all questions can be found in S2 File, along with results that were less relevant to the goals of this study. Only results and figures that pertain best to the aims of the study are included in the main text of this article, with references to appropriate sections in S2 File for more information.

### Participants

**Fig 1:**
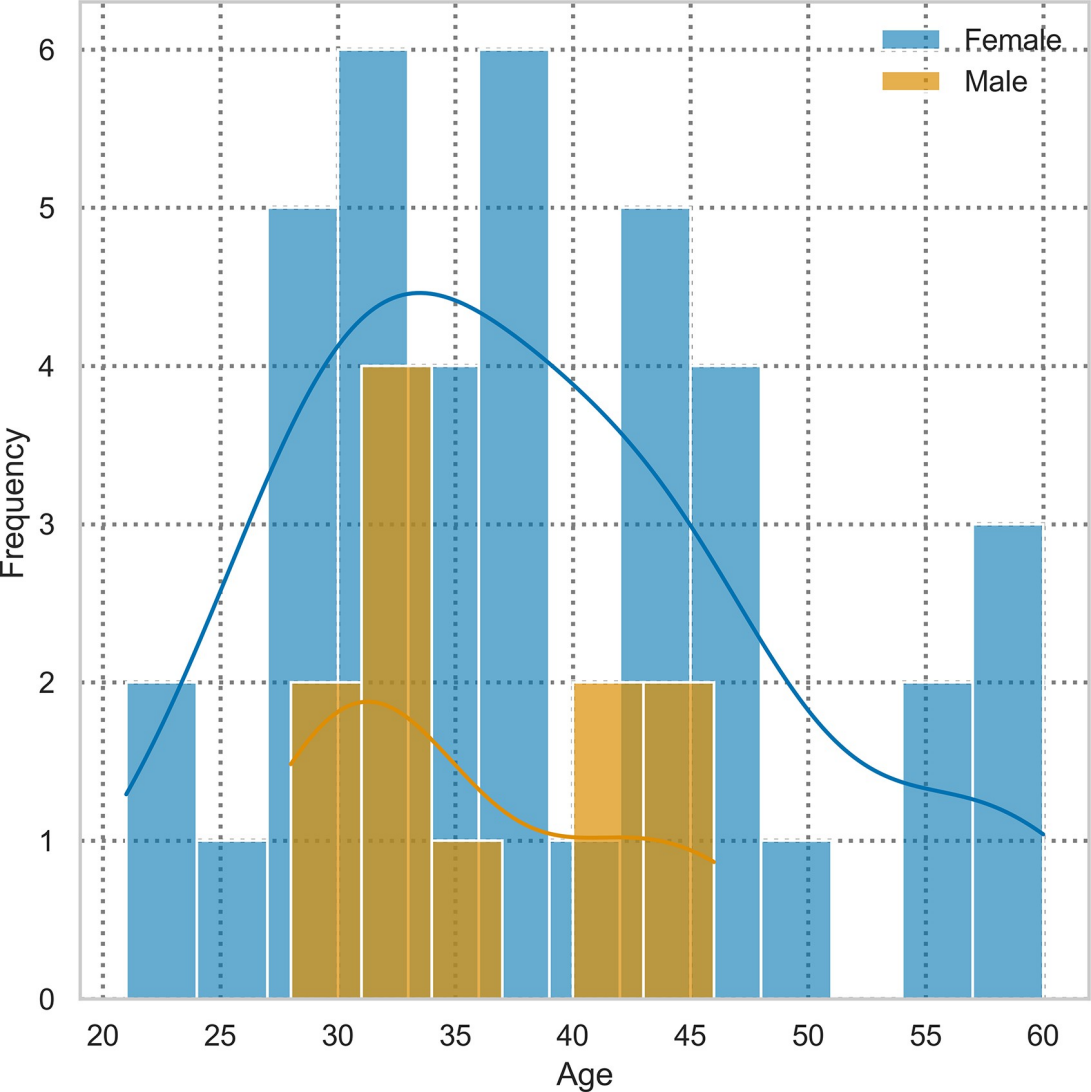
Participants’ age distribution by gender.

Age and gender distributions are reported in Fig 1. Full descriptive statistics for age are available in S2 File, section 1.1. Of the 55 participants who specified their gender, 43 (78%) were female, 12 (22%) were male, and one was non-binary (2%).

In terms of educational background (S2 File, section 1.2), one of the 52 participants (2%) had a bachelor’s degree, 27 (52%) had a master’s degree, 24 (46%) had achieved Ph.D. level. The fields in which the participants obtained their highest degrees were archaeology, biological anthropology, forensic science, biology, and design/applied arts, where a combination of up to 3 fields was indicated. The participants were also asked to self-report their current area of expertise, where 22 (43%) identified as osteoarchaeologists, while others commonly cited anthropo-archaeology, anthropology, bioarchaeology, and archaeology as fields of expertise. Geographically, anthropo-archaeology was exclusively present in France. The participants obtained their highest degrees between 1991 and 2023, and most of them graduated from their current degrees between 2016 and 2022 (S2 File, section 1.3).

All the participants currently live in Europe (S2 File, section 1.4). The best-represented country of residence was France (12 participants), followed by the UK (8 participants), and Germany (3 participants). The rest of the countries of origin (Austria, Belgium, Croatia, Cyprus, Czech Republic, Denmark, Greece, Italy, Poland, Portugal, Slovenia, Switzerland, and The Netherlands) were represented by 1 to 3 participants (S2 File). Most of the participants (n = 35, 76%) did not move to other countries for training, which means they were trained locally in their countries of origin.

Three of the respondents (5%) answered the survey in German, 17 in French (30%), and 36 in English (64%). Overall, the participants worked in twelve different languages (Danish, Czech, Dutch, English, French, German, Greek, Italian, Polish, Portuguese, Slovene, Spanish), and individual participants listed up to four different languages (S2 File, section 3.4).

There was a significant relationship between sector of activity and language, which indicated that French-speaking participants were mostly working in the public sector (χ^2^(2, N = 36) = 20.571, *p* < 0.001).

### Training and experience

Information about specific training in the analysis of cremated remains was obtained via a series of questions about the syllabus, and potential additional external training (Table 1), with space for comments (S2 File, section 2).

**Table 1:** Counts for questions regarding training. Left: number of participants that had training in cremation analysis included in their syllabus. Middle: number of participants that received training in cremation analysis outside their syllabus. Right: number of participants who received or did not receive any kind of training in cremation analysis before working with these remains.

	Syllabus		Extracurricular		Any training	
	Answers (n)	Answers (%)	Answers (n)	Answers (%)	Answers (n)	Answers (%)
**No**	27	61%	23	59%	19	43%
**Yes**	17	39%	16	41%	25	57%
**Total**	44	100%	39	100%	44	100%

Nine participants (33%) found that the time spent on cremation deposits was sufficient, while others thought more time should be spent on the subject. Time spent on cremated remains ranged from a few hours to three days in total throughout respondents’ studies. They also thought that the teaching on cremation practices and deposits was too general. Sixteen participants (35%) attended extracurricular courses or training such as summer schools, internships, or seminars on the analysis of cremated remains, while 25 participants (54%) did not participate in these kinds of training. Nineteen of the participants (43%) did not receive training in cremation analysis during their studies, nor did they attend extracurricular courses on the subject.

To evaluate the approximate level of experience of each participant with analysis of cremated material, they were asked to estimate how many cremation deposits they had analyzed in their careers and what percentage of their working time was dedicated to such analyses. Answers ranged from 2 to 3000 deposits (mean = 358, median = 60). Thirty-five (80%) out of 44 participants were actively working with cremation deposits, while six (14%) indicated they did not. Other respondents (n = 3; 7%) either acknowledged that they could encounter such remains in future projects, or that they had done this kind of analysis in the past. On average, 30% of participants’ working time was spent on the analysis of cremated remains (median = 20%). Eleven out of 41 participants (25%) indicated that 70% or more of their working time was spent on analyses of cremated material.

### Working conditions and environment

Twenty-six (59%) participants work in academia (results combined for undergraduate and postgraduate students, postdocs, research assistants, and professors). The second main sector, providing a working environment for 9 respondents (20%), was commercial. Teams of osteologists specialized in the analysis of cremated remains are small. On average, 2 people worked with cremation deposits in the labs/institutions of the respondents, although 4 participants indicated that from 5 to 10 people worked on cremation deposits in their institutions (S2 File, sections 3.1; 3.2; 3.3).

To gain a better understanding of the participants’ involvement with the cremated remains and to understand whether the analysis simultaneous to the excavation influences the analysis protocols, they were asked whether they also excavated the deposits themselves (S2 File, section 3.7). A significant fraction of participants was not engaged in the excavation (14/36 answers, 39%). On average, the least excavation is performed in academia (28%) and is more common in commercial and public sectors (52% and 65% respectively). The difference between the percentage of excavated deposits was compared between sectors where 5 or more datapoints were available [public sector, academia, and commercial sector). This difference was significant χ^2^(2, N = 33) = 6.528, *p* = 0.038. Post-hoc comparisons indicated that the excavation percent of participants from the public sector was significantly higher than that of academic participants, *p* = 0.034.

French-speaking participants excavate significantly more of the deposits they study (75% on average) when compared to respondents from other countries (35% or less on average). The difference between the percentage of excavated deposits was assessed between French-speaking and all other participants, and it was significant (*z* = -2.676, *p* = 0.007). All other participants were combined into a single group, because English usage represents a heterogeneous group of practitioners, and is not necessarily representative of a specific practice. All other groups had too little data points for the statistical analysis to be meaningful.

**Fig 2:**
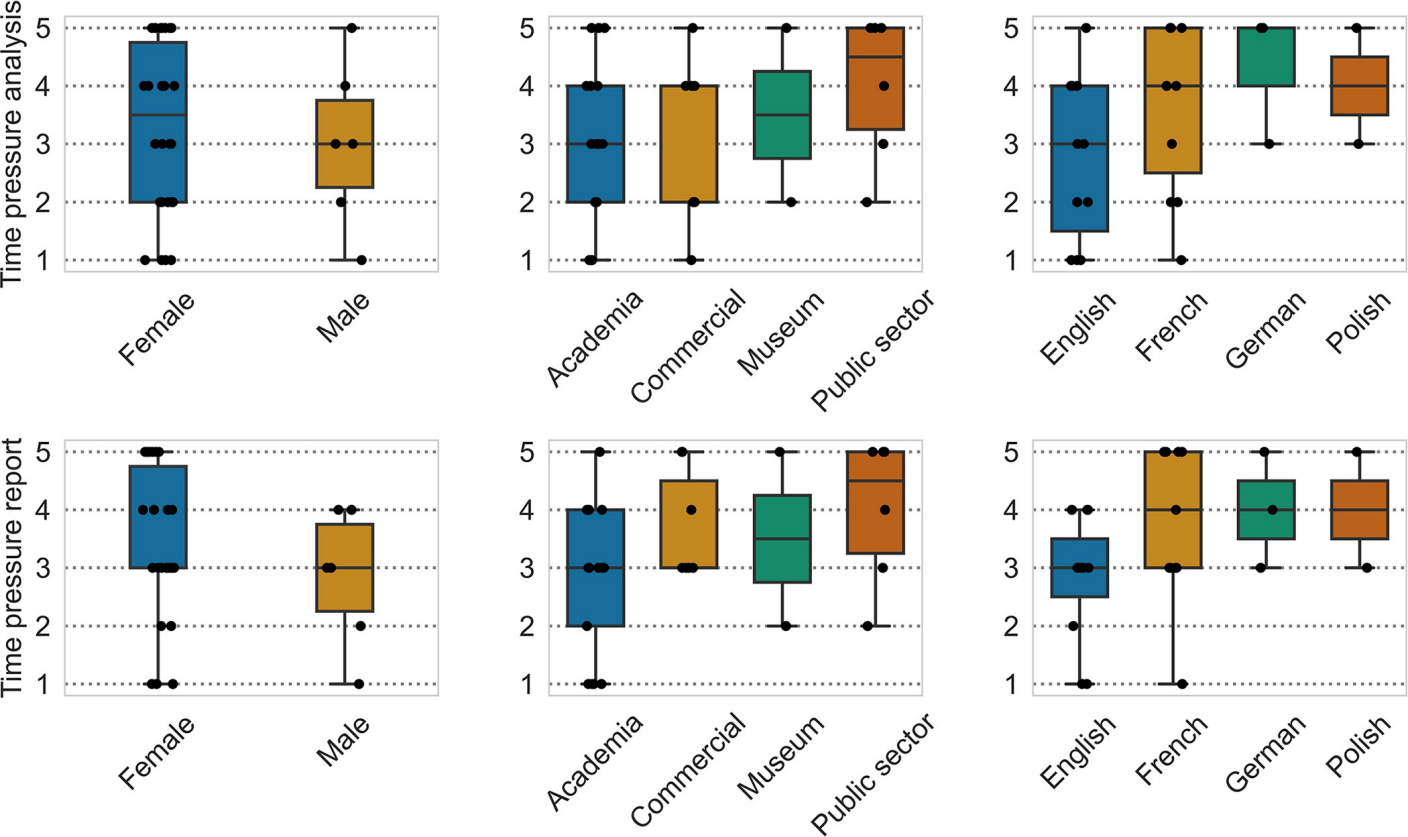
Box- and strip plots for time pressure vs. gender (left), sector (middle), and language (right). Time pressure experienced during the analysis of a cremation deposit is noted in the top row, and time pressure felt during report production is noted in the bottom row.

To assess whether time is a factor of stress for the researchers, they were asked to what degree they felt pressured, timewise, during the analysis of cremation deposits and the production of reports (S2 File, section 3.8). They were asked to evaluate this pressure on a scale from 1 to 5; 1 indicating little pressure and 5 a lot (Fig 2). The combined responses (females n = 26, males n = 7) indicate that slightly more pressure is felt to produce reports than the analysis itself. There is also a slight difference in pressure felt by gender, where male participants indicated feeling less pressure than female participants, for both analysis and reports. These tendencies, however, were not statistically significant. Participants from different sectors tended to report differences in pressure, but these were also not significant. The only statistically significant difference was that French-speaking participants feel more time pressure when producing reports (*z* = -1.957, *p* = 0.05).

When asked whether they felt pressured to produce a clear sex estimate, more than two-thirds (27/37 answers, 73%) of the participants answered that they did not feel this pressure, while the remaining third did (10/37 answers, 27%). No significant correlations between these data and gender, age, or sector were found.

### Protocols for the analysis of cremation deposits

To assess the level of standardization of the protocols, the participants were asked whether their laboratory had a specific manual for the analysis of cremated remains and/or a specific data collection form (S2 File, section 4.1). Most of the labs/institutions (22/33, 67%) did not have such a manual. Seventeen out of 32 respondents (53%) also did not have a data collection form specific to cremation deposits.

Whenever a respondent indicated that there was no form or manual, they were asked to explain their process. Very diverse answers were obtained which referred to specific manuals or protocols, descriptions of the protocol itself, or a reference to their own protocol/database. Some participants mentioned manuals or guidelines published by different institutions and researchers: *An introduction to the study of burned human skeletal remains* [10], *Analysis of human cremains* [9], *Crémation et archéologie*: *nouvelles alternatives méthodologiques en ostéologie humaine* [11], *Guidelines to the standards for recording human remains* [8], *Updated guidelines to the standards for recording human remains* [50], and the work of Henri Duday with no specific bibliographic reference. Three participants created their data collection forms or databases themselves. Examples of protocols described by the participants can be found in S2 File, section 4.1.

Participants were asked to indicate which types of data they were looking for in cremation deposits (Table 2, S2 File, section 4.2). Over 80% of participants (n = 39) assess sex, age-at-death, MNI, burning degree, and pathologies. Just over 70% of participants weigh different skeletal regions and measure the size of the largest fragment. Just over 50% of the individuals count the fragments in the deposit and weight fractions of different sizes. When under time constraint, the participants prioritize MNI over other elements of biological profile.

**Table 2:** List of things participants assessed in the cremation deposits they studied.

Assessment	Answers (n)	Answers (%)
**Sex**	32	82%
**Age-at-death**	34	87%
**MNI**	33	85%
**Burning degree**	32	82%
**Weight per size fraction**	21	54%
**Weight per skeletal region**	28	72%
**Size of the largest fragment**	28	72%
**Counting fragments**	21	54%
**Pathology**	33	85%

### Sex estimation

For sexing, 22 out of 31 participants (71%) trusted morphological methods most, three (10%) preferred a combination of metric and morphological methods, 2 (6%) did not sex cremated individuals, 2 (6%) preferred metric assessment, and 2 (6%) said that the methods they used depended on the preservation of the remains (Table 3).

**Table 3:** Methods that participants trust most.

Most trusted methods	Answers (n)	Answers (%)
**Morphological assessment**	22	71%
**Combination of morphological and metric**	3	10%
**None**	2	6%
**Metric assessment**	2	6%
**Other (depending on preservation of remains)**	2	6%
**Total**	**31**	**100%**

The respondents asserted that they trust morphological assessment “due to the lack of other credible methods”. At the same time, they acknowledged that these methods are biased due to changes in the bones due to burning. The use of metrics was disregarded by most respondents due to thermal alterations of bones which “do not allow for accurate standard measurements”, and the fact that metric estimation is only reliable when multiple elements can be used, which, they argued, is rarely the case. Participants who indicated that they did not use any sexing methods argued that they were omitting this analysis “because of the deformation of bone under the effect of heat and its high fragmentation”. Some participants used different methods depending on the preservation of the material, taking into account what they consider to be the constraints of each method (e.g., needing “seriation of frequently observed elements for population calibration” for morphological elements).

Overall, the pelvis was the most trusted skeletal region for sex assessment. The second most trusted element was the skull, and the third most trusted element was the long bones, with some people preferring hands and feet (S2 File, section 5.3). The methods that participants use in their analyses are noted in Table 4.

**Table 4:** Sex estimation methods used by the respondents.

Methods	References	Answers (n)	Answers (%)
**Morphological**	Buikstra & Ubelaker, 1994; Ferembach et al., 1979; Herrmann, 1990; Ferembach et al. 1980 [21, 22, 51, 52]	29	85%
**Metric**	Cavazzuti et al., 2019; Gonçalves, 2011; Van Vark, 1975 [36, 44, 53]	17	50%
**Lateral angle**	e.g. Gonçalves et al., 2015; Graw et al., 2005; Masotti et al., 2019 [41, 54, 55]	5	15%
**Bony labyrinth**	Osipov et al., 2013 [56]	2	6%
**Overall size and robusticity**		16	47%
**Unpublished methods**		1	3%
**Unpublished metric references**		2	6%
**Other**	Schmitt, 2008; Schmitt, 2005 [57, 58]	1	3%

Since there is a lot of variability in preservation within and between different cremation deposits, participants were asked to rank the sexually dimorphic features of the cranium and pelvis, as well as a series of published metric traits from the ones they found most often in deposits they studied, to the ones they found more rarely (S2 File, section 5.4).

**Fig 3:**
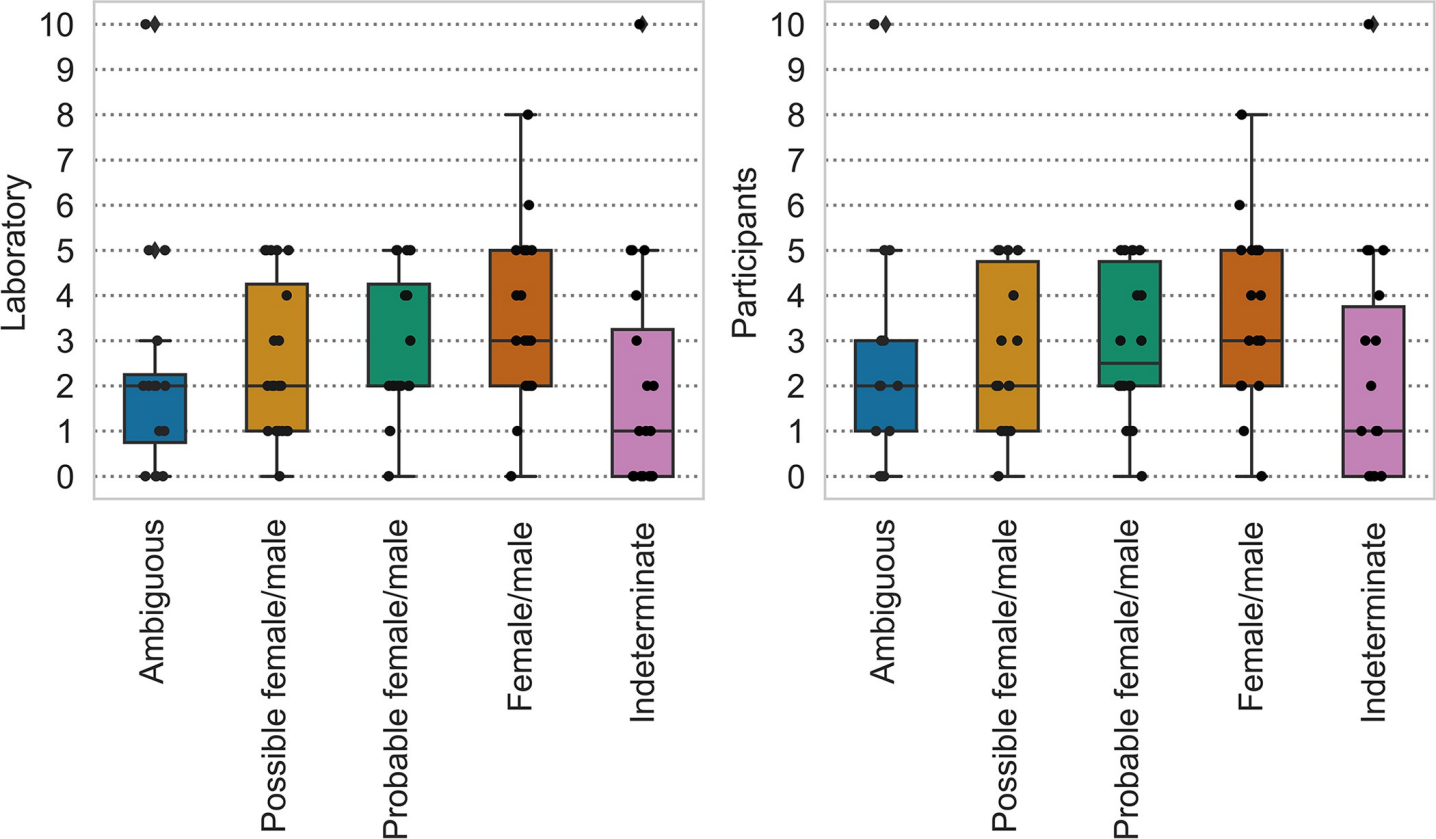
Box- and strip-plots showing the minimum number of sex-diagnostic elements that laboratories (left) and participants (right) need to establish different sex estimates.

Participants were asked whether their laboratory, or they themselves, had a rule about how many diagnostic skeletal elements are needed for sex estimation. While no clear patterns are discernible from the answers, the participants have slightly stricter rules on how many elements they will trust than a general rule for the labs, but the difference is not statistically significant (S2 File, section 5.5). The scores given by the participants are summarized in Fig 3. The differences between the scores for different estimates were significant for participants’ requirements (χ^2^(4, *N* = 12) = 17.067, *p* = 0.002). Pairwise comparisons were significant for Female/male vs. Ambiguous (*p* = 0.039) and Female/male vs. Indeterminate estimates (*p* = 0.01).

Respondents were asked whether they re-checked their work after they had done the initial analysis, and whether they re-checked it with their colleagues or other experts (S2 File, section 5.6). Three participants indicated that they always go through their work the second time, and five never do. One participant indicated that each deposit is checked at least by three separate people. They were also asked whether they use social media or other online groups to ask questions when in doubt. Only three participants indicated they were using these kinds of channels.

The types of publications in which cremation experts disseminate their results are presented in Table 5 (S2 File, section 6.1). Nearly 90% of the participants reported their findings in the form of archaeological site reports, about 50% also published in local or regional academic journals, and under 40% published in international academic journals.

**Table 5:** Types of publications where participants publish their results.

Type of publication	Answers (n)	Answers (%)
International academic journals	12	36%
Regional/local academic journals	16	48%
Archaeological site reports	29	88%
Forensic case reports	0	0%
Internal lab reports	9	27%
**Total**	**33**	**100%**

**Fig 4:**
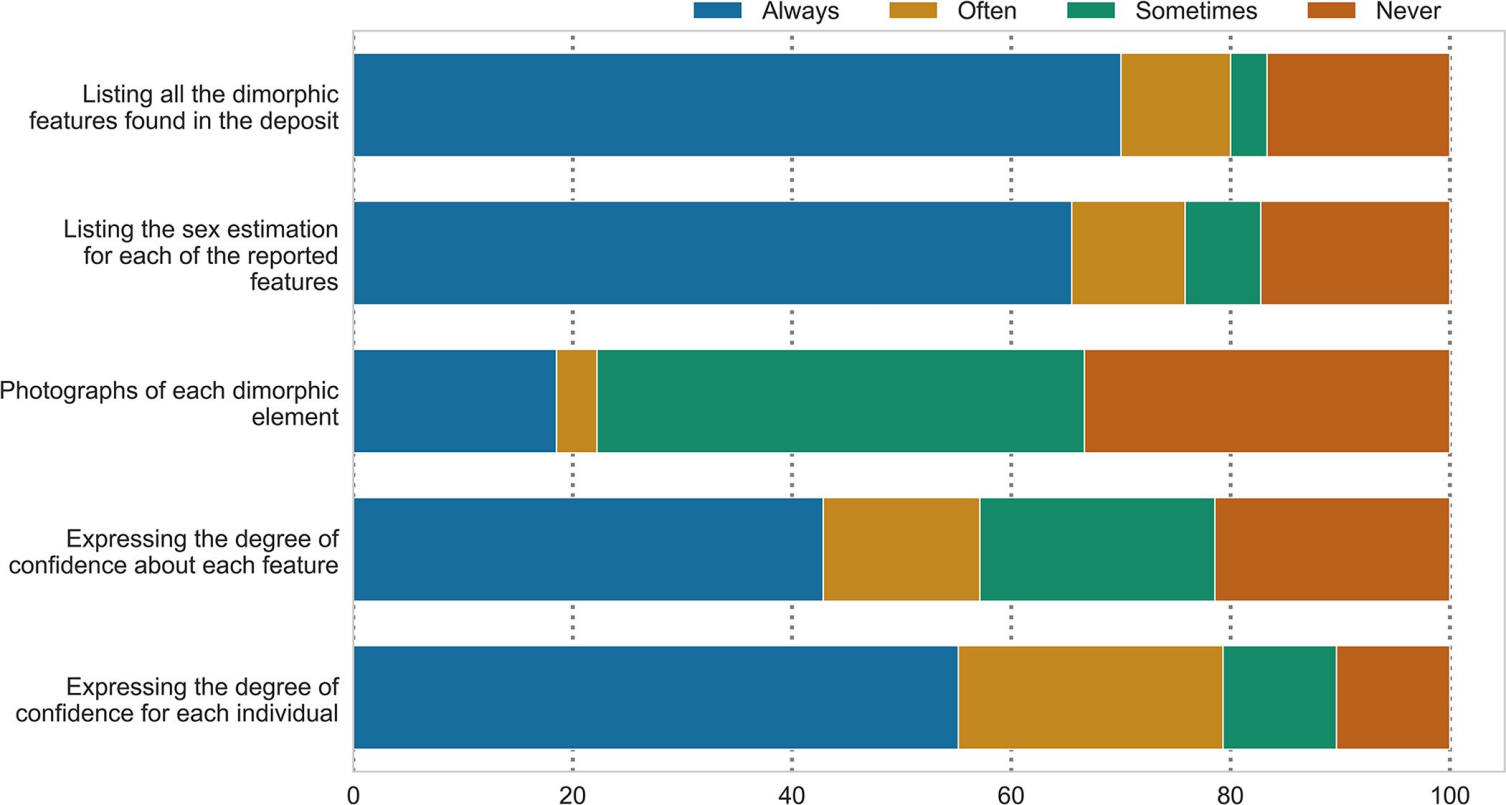
Information that participants report in publications and specialist reports regarding the sexually dimorphic elements they use in their sex estimates.

The range of information that is included in different publications is presented in Fig 4 (S2 File, section 6.2). Photographs of dimorphic elements are the least represented type of information when reporting a sex estimate, while the list of dimorphic features is commonly included.

When using multiple methods of sex estimation, the participants were asked how they decided on their final estimate. Most participants present the results of all the methods used, while the final estimate crystallizes as an average of all the used methods. Alternatively, the methods that the researcher trusts most are put forward as the most likely estimate (Table 6).

**Table 6:** Ways in which participants present sex estimation results in publications and reports.

Presenting the results of sex estimation	Answers (n)	Answers (%)
**When using multiple sex estimation methods, I present the results of each method**	13	39%
**When using multiple sex estimation methods, I only present the results that I trust the most based on experience**	6	18%
**When using multiple sex estimation methods, I present the average of all methods**	4	12%
**When using multiple sex estimation methods, I present the results of each method but elaborate a final estimate based on experience**	14	42%
**Total**	33	100%

**Fig 5:**
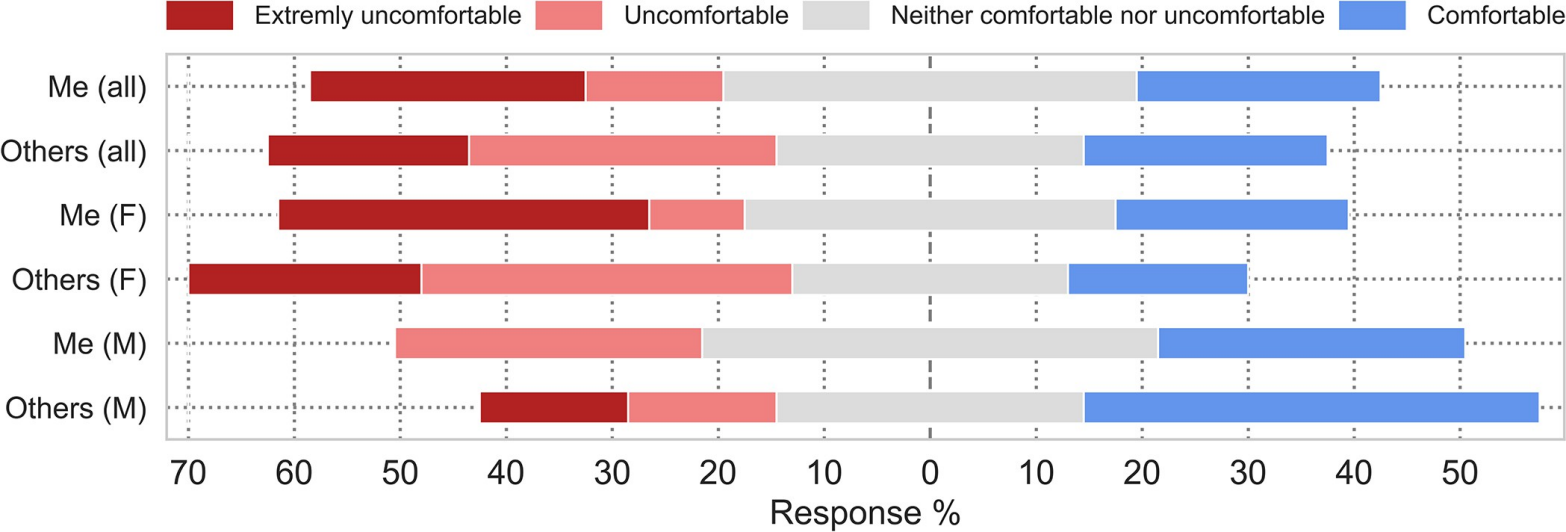
Confidence in sex estimates of cremation deposits that participants feel themselves, and confidence they expressed in the estimates of other researchers. Originally, answers were collected on a 10-point scale, but were translated into a 5-point scale by aggregating answers for better readability.

Participants reported their comfort regarding their own and others’ sex estimates (S2 File, section 5.7). The two distributions were not statistically different (*Z* = -0.303, *p* = 0.762). Different levels of confidence in their own or others’ sex estimates are reported by different genders (females n = 23, males n = 7). Based on the Likert plot (Fig 5), men trust themselves slightly less than they trust other osteologists. Women, on the other hand, tend to trust themselves slightly more than others on average, but more of them were also very unsure of their own estimates. These differences are not statistically significant (females: *Z* = -0.058, *p* = 0.954; males: *Z* = -0.552, *p* = 0.581).

Men were slightly more comfortable with sex estimation of cremation deposits for both themselves, and others, compared to female participants. While these trends can also be observed in Fig 5, the differences are not statistically significant (own estimates between genders: *z* = -1.068, *p* = 0.311 (exact. sig.); others’ estimates between genders: *z* = -1.612, *p* = 0.118 (exact sig)).

The last part of the questionnaire contained a set of open questions concerning sex estimation practices as applied to cremation deposits. Respondents were asked whether they find sex estimation important in archaeology, and why. Their answers corresponded to four different topics: 1. producing basic demographic information (biological profiles) as a base for further interpretations, 2. inferring social behavior, beliefs, and gender roles, 3. learning about funerary practices, 4. palaeodemography. While most participants found sexing “very important” (essential, critical, etc.), some of them thought that it is dependent on the research questions and that there can be “more important things to assess”, such as MNI and age groups. One participant noted that, in the past, it was one of the only aspects that could be analyzed, but that there are now “other far more interesting analyses that can be done with cremations”. German-speaking and Polish participants were especially convinced that studying large cemetery collections is essential for inferring sex patterns on a population level, as “sex determination makes little sense for individual cremations without a population context, as it is far too [unreliable]” and that in this way, “gender distribution of a population or […] a subgroup” can be inferred. Despite the perceived importance of sexing, participants concede that it is very rare to have a reliable estimate for cremation deposits, as “[numerous] studies have shown that the results obtained are systematically below the 95% confidence threshold, and that the lack of quantification and qualification of intra-population variability in archaeology leads to biases that make it impossible to apply the methods otherwise used in forensic medicine”. Respondents articulated that it is favorable to not estimate the sex for cremation deposits when not enough dimorphic elements are present, as “wrongly assessed sex can lead to false interpretations so it is better not to assess [it] in some cases”. However, some participants highlighted that they feel pressured to present a clear sex estimation by “archaeologists”, confirming that this is the main type of information they are asked to provide as expert osteologists. A worry has been expressed that some colleagues might produce sex estimation based on insufficient evidence (e.g. robusticity) because of such pressure. They also felt forced to justify why they do not provide as many estimations as other colleagues.

When asked whether they thought that methods would become available to estimate the sex of the majority of cremation deposits more confidently, three of the respondents replied negatively, arguing that dimorphic features tend to be fragmented, and that aDNA cannot be obtained from cremated bone. However, a majority of the participants are either confident or at least hopeful that methods will become available to sex a bigger proportion of cremation deposits if research continues. Hope was mostly placed in technological advances and chemical methods. Several participants suggested AMELX/Y [59], as a promising option. Other suggestions included: experimental work on individuals of known sex, where sexing would be done before and after cremation; methods tested on identified individuals/collections; increasing sample sizes and combining information from different sites where differences between different populations would be considered in the methods. One participant expressed hope that the results of this study will permit to elaborate a ‘standard workflow’.

Additional general comments consisted of different topics: 1. validation methods (methods should be validated by multiple independent researchers; they should be applicable in the field and accessible for anyone to use–not complicated statistical procedures with features with feeble dimorphism and that are not commonly found in practice; standard workflow). 2. variability of cremation deposits (preservation state is crucial to what can be done with cremation deposits, reducing the information that we can obtain to lucky coincidences) 3. sex/gender concerns (hoping that society will move away from viewing female/male as a dichotomy and superposing binary sex and genders on past populations). 4. concerns that unreliable methods are currently being used and that can lead to legal problems in forensic contexts.

## Discussion

This survey aimed to examine and reveal current practices in the analysis of cremated human remains, with an emphasis on sex estimation. The main goal was to map the situation across different countries and sectors where osteoarchaeologists and bioanthropologists work, and to examine the possibilities of standardization of the workflows and improving transparency of data, and by extension, knowledge production in the future. Additionally, pressures that participants experience related to the analysis of cremated remains and how comfortable they feel with their own and others’ sex estimates were explored.

### The place of the (micro)excavation in cremation deposit analysis

The descriptions of the protocols (S2 File, section 4.1) used by the respondents revealed that approaches to analysis and sex estimation vary significantly amongst laboratories. One of the major questions that emerged from the collected answers is the consideration of whether the excavation is deemed part of the osteological analysis, or not. Certain respondents integrated excavation into their protocol, and others did not mention it at all. Perhaps unsurprisingly, in the commercial and public sector, osteologists are more likely to excavate the deposits they study themselves, while this is rarer in academia [60]. French participants were found to excavate significantly more deposits than participants from other countries, which probably also reflects a systemic organization of archaeological work in different countries, and not individual choices of practitioners. There is also a bias in this result that reflects the fact that almost all of them were working in public sector. Despite this bias, the importance of excavation and simultaneous analysis is inherent to the “French school”, for both cremation and inhumation graves [11, 60–65]. Archaeothanatology approach allows analysts to establish a vertical distribution of anatomical regions and helps in understanding the ways in which the remains were handled between the end of the cremation and the burial of the remains [63]. Excavation in spits referred to by the “French school” practitioners [65] is of course a common practice elsewhere as well [10, 14, 66, 67], but the reality is that many osteologists study cremated material that has already been processed and cleaned [67], and are thus seldom confronted with this part of the process. Therefore, many factors external to the cremation analysis specialists dictate whether the excavation will be considered a part of the analysis or not, such as work organization and division in different institutions and countries.

### Biological profile reconstruction

Compiling the biological profile is usually the first step in the analysis of cremation deposits. Most respondents aim to collect similar types of information to achieve this, which is also reflected in the literature examined by Gonçalves and Pires [15]. The most commonly reported features of the biological profile in bioarchaeology are sex estimation, age-at-death estimation, MNI, pathology, as well as stature estimation and ancestry. The latter two are, however, rarely attempted in cremation analysis (Table 7), nor were they mentioned by any of the participants in the open questions or comments.

**Table 7:** Table with the comparison of the percentage of studies/researchers assessing different features of the biological profile in cremated remains. The data in the literature column is from Gonçalves and Pires (2017) study.

Feature	Literature (%)	Survey (%)
**Sex**	95	82
**Age**	100	87
**MNI**	96	85
**Pathology**	67	85
**Stature**	12	na
**Ancestry**	4	na

The differences in these percentages between the survey and the literature could be linked to the publication strategies of different practitioners. For instance, cases where no information about biological profile could be obtained might not be published. In fact, reliable information about the biological profile is rarely retrievable [68], as also pointed out by one of the participants regarding sexing: “[…] of the 300 cremation deposits I have studied […], I have only been able to estimate the sex of around 10 of them”.

### Assessing temperature, fragmentation, and skeleton completeness

In addition to the biological profile, many things can be measured and assessed in cremated remains, such as color and texture, weight (total weight, weight by size fractions, skeletal regions, or individual bones), size of the biggest fragment, counting the number of fragments, and types of deformations of bones [15]. These features are often used as proxies for the degree of burning (time and temperature), fragmentation, skeleton completeness, representation of different skeletal regions, inference on pre-burning conditions, and interpretation of funerary gestures [6, 20, 63, 69–71].

Color and texture of burnt bones are commonly used as proxies for burning degree, maximal temperature reached during burning, and position of the body on the pyre, but is also sometimes recorded with no specified purpose [15]. The color can be recorded using the Munsell color charts [20], or conveyed descriptively [72, 73]. Many colors and textures can be observed on a single bone fragment and an even larger range can be observed in a cremation deposit [74]. A total of 90% of published case studies [15] vs 82% of respondents in this survey noted they recorded the color of either one or multiple bones in cremation deposits. Both this survey as well as the literature review [15] make it clear that color and texture are recorded differently across the discipline, which makes comparisons between studies challenging. While color and texture are sometimes used as indicators of the burning degree [20, 71, 73], they cannot discern the exact temperatures. To maximize the pertinence of the recording of this parameter, it would be useful to agree upon a common ground for description among the experts.

As noted by Gonçalves and Pires [15], it is challenging to compare fragmentation assessments from different authors, because these elements are combined in different ways by practitioners, give different results, and can lead to different interpretations. Assessing fragmentation can be done, for instance, by sieving the remains to separate size fractions and weighing these fractions separately, to establish which one is best represented. This gives an arbitrary division between fractions for the whole cremation deposit, and it is usually done in >10mm, 5-10mm and 2-5mm fractions [67]. This approach may be associated with the ‘British school’ [15, 69], but is used widely in Europe. It is sometimes criticized [75], because sieving causes additional fragmentation of already brittle skeletal material, and according to Budzisewski [69], the shape of the opening matters for the results obtained, so it would need to be specified in the reports and papers. Sieving is absent among the French participants in the survey. The fragmentation is assessed in relative terms by calculating the index of weight between the bone flakes that are smaller than 5mm and fragments bigger than 5mm, after manual separation [63]. The areas of the deposit where the bone flakes are concentrated also help to reconstruct the ways in which remains were collected from the pyre [63]. Skeletal completeness can be based on the skeletal inventory and weights of different skeletal regions, and the mass of the cremation [9, 14]. French participants reported they assess this via the mass indices between each bone or skeletal region and their reference weights from modern crematoriums published in the literature [63, 70, 76–79]. The recording and calculation of these weight indices is done by means of a spreadsheet designed for this purpose [64]. The differences are also present in how the skeletal regions are recorded since the divisions are not uniform among practitioners. As indicated in Table 8, different skeletal regions and/or types of bone are considered during the sorting of the cremated remains. Standardized collection of this information would make the resultant data comparable. Indeed, in the interest of maximizing the value of this type of analysis, it would be worthwhile to reach a consensus on whether this should be done based on functional anatomical connections or bone types. For example, Nikita [10] suggests that, whenever possible, bones may be sorted in anatomical regions. Those elements that are not identifiable to this degree of detail could instead be sorted into broader categories (bone types) (Table 8).

**Table 8:** Comparison of different ways to sort cremated remains during the analysis, as indicated by manuals and participants.

	Author	Sorting	Additional sorting
**Manuals**	*Jaskulska*, *2020*	1. skull and dentition	
		2. trunk [spine, ribs, shoulder, and pelvic girdle)	
		3. arm and hand	
		4. leg and foot	
		5. indeterminate	
	*Nikita*, *2021*	1. cranium	1. flat
		2. pectoral girdle	2. short
		3. upper limb	3. epiphyses
		4. pelvic girdle	4. diaphyses
		5. lower limb	
		6. unidentified	
	*McKinley*, *2017*	1. skull	
		2. axial skeleton	
		3. upper limb	
		4. lower limb	
**Survey**	*Participant A*	1. skull	
		2. ribs	
		3. vertebrae	
		4. pelvis	
		5. sacrum	
		6. long bones	
		7. hand bones	
		8. feet bones	
		9 diaphyses	
		10. epiphyses	
		11. unidentified	
	*Participant B*	1. head	
		2. torso	
		3. upper limbs	
		4. lower limbs	
		5. unidentified diaphyses	
		6. epiphyses	
		7. hands and feet	
		8. rest	

More generally, it might be useful to explore the value of recording all these data, if comparisons are limited to work produced in the same institution. If data collection strategies produce more comparable data, the usefulness of such aspects as color, skeletal fragmentation, and completeness could be communicated on a larger scale. Although it is true, as Nikita [10] suggests, that analysis should be assemblage-based, some fundamental elements should be agreed upon to record cremated remains in a way that recognizes the value of scientific comparability. Cremated human remains are prone to fragmentation when subjected to repeated handling. The standardization of some aspects of data collection could eliminate the requirement for manipulation of the remains and allow for an easier overview of deposit content for sampling for future analyses (e.g. isotope analysis).

### Sexing cremated remains

In terms of methodology for sexing, morphological analysis was the absolute favorite among respondents. This confirmed the results of the literature review by Gonçalves and Pires [15], although metric methods were also more frequently used than in this survey (29% for the review vs. 3% for the survey). A total of 74% of the participants prefer using morphological methods for sex estimation, and an additional 10% indicated using a combination of morphological and metric methods, despite an array of alternative methods published in the last years [38, 42, 43]. Morphological methods are frequently employed “due to the lack of other credible methods”, even though multiple recent studies clearly indicate the biases induced by fire and the issues resultant of this for the analysis [34, 35]. The participants are aware of this problem: “we can all agree that [morphological assessment is] biased, especially if we consider the level of shrinkage/distortion and devote our observations […] to the specific populations”. Conversely, metric methods are perceived as unreliable and “not useful in cremated bone as thermal changes to bone structure do not allow for accurate standard measurements. Measurements on cremated bone can be difficult to replicate given individual changes to burnt bone that can differ based on temperature, body position, pyre, etc.”. This lack of trust might also exist due to the population-specific nature of metric methods [39, 44], and the lack of validation studies that would show sufficient reliability and reproducibility of more recently published approaches. The variability in the preservation of cremated remains likely makes it difficult to find sufficient dimorphic metric fragments to realistically assess how reliable the methods are for specific populations, and, as one respondent pointed out, “the fragmentation level of the deposits […] is usually high [so it is] very unlikely to find even minimal number of the fragments needed for [multivariate metric assessment]. I don’t believe any metric assessment that is not [multivariate] is a reliable one”. As Klales [31] noted in her survey, it is worrying that some of the participants use unpublished methods and reference collections to conduct their sex estimations even though this only concerns a small minority of the answers. It is also worrying that almost 50% of participants use robusticity as a sex estimation criterion because it is not objectively quantifiable.

The minimum number of dimorphic fragments upon which practitioners base their sex estimation does not seem to be fixed for many of the laboratories. In general, practitioners seem to base their assessments on a slightly higher number of dimorphic fragments than what is required by their institution. The answers varied significantly between different respondents, but on average the number of dimorphic fragments they used to provide what they consider a reliable estimation was between 3 and 4. Compared to the full, unburnt skeleton, where more than ten different traits can be used [21], this is a very limited number of features to base the assessment on. Another point that emerged from this question was the difference between “indeterminate” (sex cannot be estimated due to lack of traits exhibiting sex differences) and “ambiguous” (traits are present in the studied deposit but their characteristics are not indicative of any specific sex) might not be entirely clear for all the participants as indicated by the answers. Indeed, many participants thought that for “ambiguous” fewer elements are needed than for “female” or “male” estimation results.

Concerning the perceived importance of the sexing of cremated remains, respondents were confident that this information is an essential element for archaeological interpretations. However, several participants were also hopeful about moving away from the “male-female” dichotomy in bioarchaeology. Respondents were generally optimistic about the future of sexing cremated individuals, but most placed their trust in technological developments, rather than in the improvement of the currently available osteological methods.

### Time pressure for analysis and confidence in sexing

For the first time, information has been collected on whether cremation experts feel time pressure when analyzing cremation deposits, or during report production. These questions were included because they have the potential to reveal factors of influence contributing to the non-standardization of protocols, as well as the quality of the data produced, even if they are sometimes difficult to quantify. Reports generated more pressure in terms of timing than the analysis itself. Despite a significant gender bias in the numbers of male and female participants, the results indicate that men felt less pressure than women for both parameters. This result is not surprising, as time pressures are more frequently reported in women than men, in occupation [80] and leisure [81]. In terms of the pressure to produce a clear sex estimation, 70% of respondents felt no pressure, which is a positive result. Conversely, 30% of the participants felt this type of pressure, a worrying result given all the difficulties related to analyzing cremated remains.

The degree of confidence in the estimations produced by participants themselves, and in those of other colleagues was only assessed for sexing. These questions were included to assess how practitioners felt about their own estimates and whether this was correlated with their level of experience. Comfort scores vary greatly between experts (Fig 5). Some hesitate little to assign sex to cremated remains, but others need more elements to feel secure in their assessment. While men seem to be slightly more comfortable with their sex estimates than women, it is interesting that they also trust the estimates of other people more than their own. Women more frequently indicated that they are not comfortable with other people’s sex estimation, while they are slightly more comfortable with their own. The intensity of distrust in sex estimates generally is higher in women (‘extremely uncomfortable’ with their own estimates vs. ‘not comfortable’ with the estimates of others). The gender gap in confidence is a well-known phenomenon in academia and has been recognized more widely as well [82, 83]. Despite relatively low confidence, re-checking of the results is not omnipresent among the participants, and only 20% reach out to colleagues via social networks and mailing lists.

### Diversity in analyses of cremated remains

The study of cremated remains is marked by a high degree of variability [15] that mirrors the variability of the cremation rite and subsequent processes [6, 65, 84]. Highlighting the factors leading to this inconsistency may provide a better understanding of what constitutes current practice.

The first contributing factor is training. As many as 41% of the participants did not receive any specific training in the analysis of cremated remains. This indicates that they are largely self-trained. The survey highlights that syllabuses have different approaches, but there is a general tendency for university programs to neglect discussions of cremation practices, and the analysis of cremation deposits. Consequently, osteoarchaeologists interested in cremated remains need to seek out additional advice or training from more experienced specialists.

The second factor is the lack of laboratory and institutional guidelines on how to proceed with a cremation deposit. Over half of the respondents’ laboratories lacked manuals and standardized data collection forms specific to cremated remains. The practitioners are therefore left to their own devices and often rely on published guidelines [8–10, 13, 63], which contain varying degrees of precision on how the deposits should be studied, recorded, and reported on.

The third factor arises from the different traditions or ‘schools’, as already highlighted by Gonçalves and Pires [15]. They found that French, Spanish, and English traditions exist, while a German tradition is less recognizable [15]. This survey confirmed the presence of a French ‘school’, due to the relative overrepresentation of French participants. The two major elements of this practice are the excavation methodology in spits [65], and the relative weights of different skeletal regions with reference to modern remains from crematoria. The latter information is recorded in spreadsheets that calculate the weight indices for bones and skeletal regions to reconstruct the completeness of the skeleton and to infer details about the post-cremation handling of the remains [61, 63]. Although much less prominent, a tendency was discerned for German and Polish-speaking participants to insist on the importance of seriation and population-wide data collection for sex estimation. It is likely that other regional traditions exist, but the results of this survey do not allow us to discern them.

Several researchers proposed “standard workflows” in their guidelines [9, 10, 14], as well as conference talks (Silva, 2023, EAA 2023, session 401). However, despite their accessibility (open source online), there is no universal preference, and different practices and regional/linguistic traditions persist. While their goals are largely similar, they put their emphasis on different details and collect data differently. It may be illusory to expect that one protocol will satisfy all practitioners in private, public, heritage, and academic settings. Indeed, they work with different contexts, limitations, financial pressures, and time constraints. Some experts might consider certain types of data a priority over others, depending on their research questions, or those imposed on them by the institution or companies they work for. Finally, most of the respondents worked in settings where they were either the only osteologist, or one of two. Only four respondents were part of a bigger team (5–10 specialists). This gives practitioners a great degree of freedom when it comes to choices in protocols and might also add to their uncertainty.

In discussions on standardization, an important aspect of conveying the information of the analyses is often overlooked: the reporting of the results. While the reporting was only assessed in detail for sex estimation, the disparities might also be true for other parameters of the biological profile, such as age-at-death, MNI, fragmentation, and temperature. Individual reporting choices are almost certainly also an important factor for the lack of data comparability, as they can hide how the data were obtained in the first place. There are no clear rules on how to report sex estimation and other results in the literature. Conveying the data in a transparent way that can be judged by peers is essential.

### Future work

While striving towards a standardization of protocols is a good thing, it might not be entirely possible to achieve ‘the one and only’ workflow for all the reasons listed above. Additionally, since propositions for these workflows already exist, it would be counterproductive to keep proposing new ‘universal workflows’. Instead, given the diversity in the field, an international, interactive consensus could perhaps be useful to discuss which existent guidelines should be followed, and perhaps improved in an inclusive manner, with experts from all professional backgrounds (preventive, commercial, academic) and countries having the opportunity to express why some protocols are more appropriate for their practice. The recommendations could then be proposed for the wider community of experts as best practice.

At the same time, the aim should be to achieve more transparency on how data are obtained, and which choices are made in the process. This can be done by agreeing on minimal standards in the reporting of the results. At the very least, this would reveal the fundamental elements that contribute to published sex and age-at-death estimations, MNI, and fragmentation assessments. The first step towards more transparency is to encourage the publication of images of the deposit and other relevant details. As already emphasized by Depierre [61], a full list of diagnostic elements with photographs and reasoning on which estimations are based on should be included in reports, as well as academic literature, along with information on the methods applied for specific elements. In this way, the evaluation of the quantity and quality of the material that the assessments were based on would be possible. As there is hardly space for such reports in the structure of the academic papers, they could be a part of the supplementary information. Ultimately, more intensive teaching on cremation analysis should be included in university syllabuses. This would allow practitioners to access basic information on the possibilities and limitations of the discipline. It would also be useful to outline different traditions and their core characteristics so that osteologists-in-training become familiar with the state-of-the-art methods in cremation research.

In terms of sex estimation, it should be made clear to the wider (archaeological) community that this particular information is difficult to obtain, in order to manage expectations and mediate pressures that could produce unreliable results. Indeed, the findings of this study demonstrate that none of the experts feel very comfortable with assigning sex to cremation deposits, and that this comfort is only very weakly correlated with experience. It would also be useful to invest time and efforts in validation studies for recently published methods, to increase the level of trust among osteoarchaeologists in these approaches. While novel research is essential, it can only find its rightful place in practice if there is sufficient evidence that the proposed methods are reliable, reproducible, and possible to implement for many practitioners.

### Biases and limitations

The present survey was directed at experts working with cremated remains in different countries, a variety of different institutions and companies, and it is subject to several biases. By disseminating this survey via the authors’ professional networks, people who received an invitation to participate were largely from academic circles, which is also reflected in the high number of participants from this sector (60%). Moreover, due to the degree of specialization necessary when working with human skeletal remains, it is unsurprising that most of the participants hold a master’s or a PhD degree in either archaeology, bioarchaeology, biological anthropology, biology, or forensic science. The gender ratio of respondents is strongly skewed towards women, which likely reflects the overrepresentation of women researchers and workers in the field of (bio)archaeology in certain European countries [85]. This imbalance needs to be considered, especially in the context of questions about the perceived pressure in analysis and report writing, as well as confidence (expressed as the degree of comfort in assigning sex estimations to cremated remains in this study). Furthermore, all authors work in academia, which might have biased the questions to speak more to an academic audience. The overrepresentation of French participants is likely due to dissemination via the “Société préhistorique française” that has a large membership. The representation of participants from different countries and the absence of answers from some countries means that our results might not represent the full picture of practices everywhere in Europe.

A wider response was hoped for, but several factors influenced the limited number of respondents: as pointed out by two participants, the number of people working in the field is quite small and the complete anonymity of participants cannot be guaranteed–perhaps some were not willing to take this risk, although the results are only used for scientific purposes. Secondly, the questionnaire was time-consuming, which might have dissuaded some people from finishing. Remuneration could perhaps partly address this problem and should be considered for further studies.

## Conclusions

This study’s aim was to map the practices of analysis of cremated remains, with an emphasis on the issues linked to sex estimation. Despite a limited number of responses, some interesting findings emerged which open up directions for further work.

Protocols of analyses vary between practitioners and laboratories, and the main reasons discerned for these differences were the insufficient and disparate training of new osteologists, the lack of institutional guidelines on recommended protocols, and different regional traditions. The absence of consensus on a workflow that would produce comparable data is also a major factor of these different approaches.

In terms of sex estimation, the majority of participants did not feel pressure to produce clear sex estimates. Conversely, 30% of participants did feel this pressure, which does represent the danger of basing sex estimates on insufficient evidence. Morphological methods are generally the preferred way of estimating sex, despite the awareness that in cremated remains, this method is biased due to thermal alterations of bone. Metric sex estimations are widely distrusted by many of the colleagues who participated in the survey.

The suggestions that can be formulated based on these data are to: 1. increase the time dedicated to studying cremated remains in university syllabuses, 2. formalize analysis protocols in the institutional context, 3. work in an inclusive way to make as many data categories as possible comparable between practitioners and laboratories (e.g. the way the color of the bones is recorded, which bone categories and anatomical regions should be widely used to sort the bones into, whether to sieve the remains), 4. outline the standards required for reporting findings, 5. encourage the production and publication of validation for new methods for sex estimation, and 6. diminish the pressure on cremation analysis experts to provide clear estimates by making the wider archaeological community aware of the limitations these remains represent.

## Supporting information

S1 File Questionnaire. (PDF)

S2 File Descriptive statistics, supporting information. (PDF)
