# Supplementary material for: Practitioner preferences in the analysis of cremation deposits in archaeology and biological anthropology: An overview of current osteoarchaeological practices with a focus on sex estimation
Source: PLoS One. 2024 Dec 2;19(12):e0310380. doi: 10.1371/journal.pone.0310380 (PMC11611176; doi:10.1371/journal.pone.0310380)
Supplement: S1 File — (PDF) [file pone.0310380.s001.pdf]

Sex estimation practices of cremated remains among archaeologists and biological anthropologists

Whether performed upon cremated or unburnt human remains sex estimation is considered a fundamental element for establishing biological profiles in bioarchaeology. Osteological data provides us with a basis for further research on palaeodemography, past socio-cultural practices, and palaeopathology. Despite the fundamental nature of biological sex within archaeological datasets, we rarely question how this data was obtained. While there is widespread confidence among archaeologists and anthropologists in various methods, there is, to our knowledge, no clear, international consensus on a protocol for sexing cremations. This survey aims to identify patterns in sex estimation practices among experts working with cremated human remains from archaeological contexts. Whilst a literature review might be used to assess these practices, it is, in our opinion, not as exhaustive. This is because it is difficult to access grey literature, often published in the regional languages of the practitioners, and used as a primary source of information for later publications in peer-reviewed journals. With the questionnaire, we would like to give practitioners the opportunity to communicate their research practice. This will allow to identify the differences and similarities in protocols among different geographical regions and training traditions, and most importantly, start looking for solutions for the standardization of sex estimation protocols for cremation deposits in the archaeological research community. This study is a part of the PhD project looking into sex estimation in cremated remains. The results will be published in the form of articles in relevant academic journals. Additionally, the results will be disseminated at scientific conferences.

We kindly invite you to participate in this survey on sex estimation practices of cremated human remains among archaeologists and biological anthropologists. The survey takes approximately 30 minutes to complete. You can stop at any time and save your results to come back to it later. Your participation is voluntary: you are not obliged to take part and if you refuse, this will have no consequences for you. Take enough time to decide whether you want to participate. Any questions or concerns can be communicated to the researchers via email. You can stop your participation at any time, and you do not have to give a reason for doing so. Below you can find more information about the study.

Contact details

Marta Hlad

AMGC, Vrije Universiteit Brussel (VUB)

Email: meta.sexing@protonmail.com

There are 49 questions in this survey.

Participants

The first part of this survey concerns the information on the participants, such as age, gender, and education background.

Gender

Check all that apply

Please choose **all** that apply:

- [ ] Female*
- [ ] Male*
- [ ] Non-binary
- [ ] Rather not say
- [ ] Other:

By Male* and Female* we mean all individuals who identify with either gender, including cis- and transgender individuals.

Age

Only numbers may be entered in this field.

Please write your answer here:

Education level

Choose one of the following answers

Please choose **only one** of the following:

- ○ Bachelor
- ○ Masters
- ○ Ph.D
- ○ Other

Please select the highest degree obtained.

What field is your highest degree from?

❗ Check all that apply

Please choose **all** that apply:

- [ ] Archaeology
- [ ] Anthropology (biological / physical)
- [ ] Forensic science
- [ ] Biology
- [ ] Other:

In what year did you obtain your highest degree?

❗ Only numbers may be entered in this field.

Please write your answer here:

What is you main area of expertise (e.g archaeology, biology, osteoarchaeology, anthropology, etc.)?

Please write your answer here:

Country of origin

Please write your answer here:

Country of residence

Please write your answer here:

Training in the analysis of cremated remains

This part concerns the training you received in terms of the analysis of cremated remains, both as a part of training syllabus or in different external courses.

Country of training

Please write your answer here:

Please select the country where you were trained in archaeological/anthropological analysis of cremated human remains

Institution of training

Please write your answer here:

Please specify the institution where you were trained.

Did your training syllabus include a specific course on the analysis of cremated remains?

Please choose **only one** of the following:

- ◯ Yes
- ◯ No
- ◯ Other

Make a comment on your choice here:

You can provide a description of the course in the comment, with information such as duration and content of the course.

If your syllabus did include training on cremation remains, do you think enough time was spent on this topic?

Please choose **only one** of the following:

- Yes
- No
- Other

Make a comment on your choice here:

Did you attend a specialized course on the analysis of cremated remains, such as summer school, short courses, internally organized seminars, etc.?

Please choose **only one** of the following:

- ◯ Yes
- ◯ No
- ◯ Other

Make a comment on your choice here:

You can provide a description of the course in the comment, with information such as duration and content of the course.

Approximately how many cremation deposits have you analyzed in your career?

Please write your answer here:

Working environment

This part of the survey aims to collect data on working environments that archaeologists and anthropologists studying cremations are part of.

What is your current professional situation?

Please choose **all** that apply:

- [ ] Bachelor student
- [ ] Master student
- [ ] Ph.D student
- [ ] Postdoc
- [ ] Research assistant
- [ ] Professor
- [ ] Employee in a comercial compagny
- [ ] Retired
- [ ] Other: ______

When does your current contract end?

Please choose **only one** of the following:

- ( ) < 1 year
- ( ) < 3 years
- ( ) < 5 years
- ( ) 5 - 10 years
- ( ) Indeterminate
- ( ) Other ______

What institution are you currently affiliated to?

Please write your answer here:

______

What is the main language you use at work?

Please write your answer here:

Is the osteological analysis of cremated remains a part of your current job?

Please choose **only one** of the following:

- Yes
- No
- Other

Make a comment on your choice here:

If the answer is no or other, could you please specify your past/present experience with osteological analysis of cremated remains?

Approximately what percentage of your working time is related to cremations?

Please write your answer here:

How many people in your lab, including you, do osteology on cremations?

Please write your answer here:

What other types of analyses does your lab perform on cremated remains?

Please choose all that apply and provide a comment:

- [ ] Isotope analysis
- [ ] 14C dating
- [ ] Spectroscopy

Other:

Apart from osteology, do you personally do other types of analysis on cremations?

Please choose **only one** of the following:

- Yes
- No
- Other

What percentage of the cremation deposits that you analyze do you also excavate yourself (either in the field or micro-excavation in the lab)?

Please write your answer here:

Are you under any type of time constraints per analyzed cremation deposit or time pressure to produce a report?

Please choose the appropriate response for each item:

	1	2	3	4	5
**Amount of time per cremation deposit**	○	○	○	○	○
**Writting reports on 1 or more deposits**	○	○	○	○	○

On a scale from 1 to 5 where 1 is little and 5 is a lot, how much time pressure is put on you to complete the following tasks?

Do you feel under pressure to produce clear sex estimations when analyzing cremation collections?

Please choose **only one** of the following:

- Yes
- No
- Other

Cremation analysis protocol

In this part, we ask you for information about the cremation analysis protocols you use in your lab.

Is there an internal lab manual specifically for cremations at your lab?

Please choose **only one** of the following:

- Yes
- No
- Other

Is there a data collection form specifically for cremations in your lab?

Please choose **only one** of the following:

- Yes
- No
- Other

If you answered yes to the previous question, could you please upload a lab form that you use for the analysis of cremated remains?

Kindly attach the aforementioned documents along with the survey

If there is no form, do you have your own protocol that is formalized in any way? Could you describe the process of analyzing one cremation deposit?

Please write your answer here:

What do you assess in cremation deposits?

Please choose all that apply and provide a comment:

- [ ] Sex
- [ ] Age
- [ ] Minimum number of individuals
- [ ] Burning degree (visual assessment)
- [ ] Weights of different size fractions
- [ ] Weigths of different skeletal categories
- [ ] Maximum fragment size
- [ ] Number of fragments
- [ ] Pathology

Other:

If / when under a time constraint, what parts of the analysis do you prioritize? Please sort the answers from the top priority on the top to the lowest priority on the bottom.

Please number each box in order of preference from 1 to 7

[ ] Sex

[ ] Age

[ ] Minimum number of individuals

[ ] Burning degree

[ ] Weights of different size fractions

[ ] Weights of skeletal categories

[ ] Maximum fragment size

Sex assessment

Which type of method do you think is the most reliable for sex estimation?

Please choose **only one** of the following:

- ◯ Morphological assessment (qualitative)
- ◯ Metric assessment (quantitative)
- ◯ Other

Make a comment on your choice here:

Which areas of the skeleton do you think are the most reliable for sex estimation? Please sort the answers from the most reliable on the top to the least reliable on the bottom.

Please number each box in order of preference from 1 to 5

- ☐ Skull
- ☐ Pelvis
- ☐ Long bones
- ☐ Hands / Feet
- ☐ Other

Which methods and characteristics do you use to estimate sex in cremation deposits?

Please choose all that apply and provide a comment:

- [ ] Morphological and metric methos (e.g. Buikstra and Ubelaker 1994; WEA 1980)
- [ ] Metric methods for cremated remains (e.g. Cavazutti et al. 2019; Van Vark 1979, Gonçalves et al. 2013))
- [ ] Lateral angle of acustic meatus (e.g. Masotti et al. 2019; Gonçalves et al. 2015; Graw et al. 2005)
- [ ] Cochlear measurements and shape (e.g. Osipov et al. 2013)
- [ ] Overall size and robusticity
- [ ] Method(s) that me or my lab developed (unpublished)
- [ ] Metric methods based on my own reference collections (unpublished)

Other:

Which of the sexually dimorphic morphological features of the pelvis and cranium do you most often find in cremation deposits you study? Please sort them in order from the most commonly found to the least commonly found.

Please number each box in order of preference from 1 to 11

[ ] Sciatic notch
[ ] Subpubic angle
[ ] Ventral arc
[ ] Pubic bone length
[ ] Supraorbital ridge
[ ] Mastoid process
[ ] Posterior zygomatic arch
[ ] Nuchal crest/Occipital protuberance
[ ] Mental eminence
[ ] Supraorbital margin
[ ] Gonial angle

Which of the skeletal elements with sexually dimorphic metric features do you find most often in cremation deposits you study? Please sort them from the most commonly found on the top to the least commonly found on the bottom.

Please number each box in order of preference from 1 to 16

- [ ] Mandible
- [ ] Axis (dens)
- [ ] Scapula (glenoid fossa)
- [ ] Humerus (proximal)
- [ ] Humerus (distal)
- [ ] Radius (proximal)
- [ ] Lunate
- [ ] Scaphoid
- [ ] Hamate
- [ ] Femur (head)
- [ ] Patella
- [ ] Talus
- [ ] Navicular
- [ ] First metatarsal
- [ ] Navicular
- [ ] Other

Does your lab require a minimum number of dimorphic elements to establish a sex estimation? Please choose how many by selecting the corresponding number.

Please choose the appropriate response for each item:

	0	1	2	3	4	5	6	7	8	9	10	>10
**Ambiguous**	○	○	○	○	○	○	○	○	○	○	○	○
**Possible female/Possible male**	○	○	○	○	○	○	○	○	○	○	○	○
**Probable female/Probable male**	○	○	○	○	○	○	○	○	○	○	○	○
**Female/Male**	○	○	○	○	○	○	○	○	○	○	○	○
**Indeterminate**	○	○	○	○	○	○	○	○	○	○	○	○

Do you personally have a clear rule about a minimum number of dimorphic elements to establish a sex estimation? Please choose how many by selecting the corresponding number.

Please choose the appropriate response for each item:

	0	1	2	3	4	5	6	7	8	9	10	>10
**Ambiguous**	○	○	○	○	○	○	○	○	○	○	○	○
**Possible female/Possible male**	○	○	○	○	○	○	○	○	○	○	○	○
**Probable female/Probable male**	○	○	○	○	○	○	○	○	○	○	○	○
**Female/Male**	○	○	○	○	○	○	○	○	○	○	○	○
**Indeterminate**	○	○	○	○	○	○	○	○	○	○	○	○

Do you have a habit of going through each studied collection a 2nd time to double-check your estimations?

Please choose **only one** of the following:

- Never
- Sometimes
- Often
- Always
- Other

If working in a team with other osteoarchaeologists, how often do you ask for an opinion of a colleague to double-check your conclusions on sex estimation?

Please choose **only one** of the following:

- Never
- Sometimes
- Often
- Always
- Other

Reporting

This part of the survey is about how you report your results of cremation analysis, particularly the sex estimation.

Where do you report the results of your cremation analyses?

Please choose **all** that apply:

- ☐ International academic journals
- ☐ Regional / local academic journals
- ☐ Archaeological site reports / specialist reports
- ☐ Forensic case reports
- ☐ Internal lab reports
- ☐ Other: ____________

How do you report the sex estimation in these publications?

Please choose the appropriate response for each item:

	Never	Sometimes	Often	Always
**Listing all the dimorphic features found in the deposit**	○	○	○	○
**Listing the sex estimation for each of the reported features**	○	○	○	○
**Photographs of each dimorphic element**	○	○	○	○
**Expressing the degree of confidence about each feature**	○	○	○	○
**Espressing the degree of confidence for each individual**	○	○	○	○

When using multiple sex estimation methods, how do you report the results?

Please choose all that apply and provide a comment:

- [ ] I present the results of each method
- [ ] I only present the results that I trust the most based on experience
- [ ] I present the average of all methods
- [ ] I present the resutls of each method but elaborate a final estimaton based on experience

Other:

Thoughts and comments

This set of questions is about your opinion on the sex estimation of cremations.

How comfortable are you with:

Please choose the appropriate response for each item:

	1: Extremly comfortable	2	3	4	5	6	7	8	9	10: Extremly uncomfor
**Assigning a clear sex estimation to a cremation deposit**	◯	◯	◯	◯	◯	◯	◯	◯	◯	◯
**Trusting other researcher's sex estimations of cremation depostis**	◯	◯	◯	◯	◯	◯	◯	◯	◯	◯

Is estimating sex of cremated deposits important to you and why?

Please write your answer here:

In your opinion, is the sex estimation of cremation deposits essential to archaeological research? Please elaborate.

Please write your answer here:

Do you think we will find a way to reliably estimate sex of the majority of cremation deposits in the future?

Please write your answer here:

Do you have any additional thoughts and comments on the sex estimation of cremated remains, or on this survey?

Please write your answer here:

Thank you very much for completing this survey. If you are interested in the results that will come out of it please follow the Brussels Bioarchaeology Lab on Twitter (@BrusselsBioarch), check the website https://www.bb-lab.be/, or email meta.sexing@protonmail.com.

Submit your survey.
Thank you for completing this survey.
