# Supplementary material for: Practitioner preferences in the analysis of cremation deposits in archaeology and biological anthropology: An overview of current osteoarchaeological practices with a focus on sex estimation
Source: PLoS One. 2024 Dec 2;19(12):e0310380. doi: 10.1371/journal.pone.0310380 (PMC11611176; doi:10.1371/journal.pone.0310380)
Supplement: S2 File — (PDF) [file pone.0310380.s002.pdf]

# Summary and descriptive statistics

## 1.Participants

### 1.1 Age and gender

Age descriptive statistics are reported Table 1. The values in the age column are not normally distributed (Table 2). Gender counts are presented in Table 3.

*Table 1: Descriptive statistics for age.*

| Age | | Statistic | Std. Error |
|---|---|---|---|
| **Mean** | | 37.25 | 1.295 |
| **95% Confidence Interval for Mean** | **Lower Bound** | 34.65 | |
| | **Upper Bound** | 39.84 | |
| **5% Trimmed Mean** | | 36.86 | |
| **Median** | | 35.00 | |
| **Variance** | | 88.919 | |
| **Std. Deviation** | | 9.430 | |
| **Minimum** | | 21 | |
| **Maximum** | | 60 | |
| **Range** | | 39 | |
| **Interquartile Range** | | 14 | |
| **Skewness** | | .706 | .327 |
| **Kurtosis** | | -.015 | .644 |

*Table 2: Normality tests for age.*

| Kolmogorov-Smirnov | | | Shapiro-Wilk | | |
|---|---|---|---|---|---|
| Statistic | df | Sig. | Statistic | df | Sig. |
| .126 | 53 | *.035* | .944 | 53 | *.016* |

*Table 3: Counts for gender.*

| Gender | Count |
|---|---|
| **Female** | 41 |
| **Male** | 11 |
| **Non-binary** | 1 |

A Mann-Whitney U test was performed to check whether there was a significant difference in age distribution between genders. Only the difference between males and females was computed, as only one person was non-binary. The difference in age distribution between females and males was not statistically significant ($z$ = -0.653, $p$ = .513). The data is also summarized in the Figure 1.

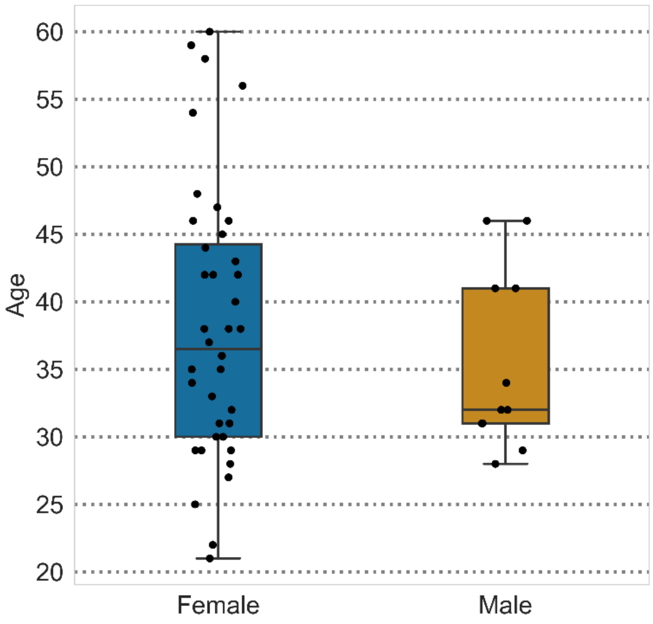


*Figure 1: Box- and strip plots representing age distributions by gender.*

## 1.2 Degrees and areas of expertise

In terms of education, most of the participants had a master's or a Ph.D. degree (Table 4). The difference in the degrees and genders was compared using Chi-Square test, and was not statistically significant ($\chi2$ (3, $N$ = 52) = 1.038, $p$ = .792). The relationship between the degree and sector was also not statistically significant ($\chi2$ (4, $N$ = 36) = 7.420, $p$ = .115).

*Table 4: Counts of different degrees obtained by the participants.*

| Degree | Answers (n) | Answers (%) |
|---|---|---|
| **Bachelor** | 1 | 2% |
| **Master** | 27 | 52% |
| **Ph.D.** | 24 | 46% |
| **Total** | 52 | 100% |

Fields in which participants obtained their degrees and participants' main areas of expertise are listed in Tables 5 and 6.

*Table 5: Counts and percentages of the subjects in which participants obtained their degrees.*

| Degree fields | Answers (n) | Answers (%) |
|---|---|---|
| **Biological anthropology** | 17 | 31% |
| **Archaeology** | 17 | 31% |
| **Archaeology and biological anthropology** | 15 | 28% |
| **Biological anthropology and biology** | 2 | 4% |
| **Design and applied arts** | 1 | 2% |
| **Archaeology, biological anthropology, and biology** | 1 | 2% |
| **Archaeology, biological anthropology, and forensic science** | 1 | 2% |
| **Total** | 54 | 100% |

*Table 6: Counts of participants' main areas of expertise.*

| Main area of expertise | Answers (n) | Answers (%) |
|---|---|---|
| Osteoarchaeology | 22 | 42% |
| Bioarchaeology | 5 | 9% |
| Anthropo-archaeology | 5 | 9% |
| Archaeology | 5 | 9% |
| Anthropology | 5 | 9% |
| Biological anthropology | 3 | 6% |
| Biomolecular archaeology | 1 | 2% |
| Archaeology and Anthropology | 1 | 2% |
| Palaeoanthropology | 1 | 2% |
| Archaeological Sciences | 1 | 2% |
| Evolutionary anthropology | 1 | 2% |
| Archaeology and Bioarchaeology | 1 | 2% |
| Archaeothanatology | 1 | 2% |
| Battlefield archaeology | 1 | 2% |
| Total | 53 | 100% |

## 1.3 Year of graduation

Summary statistics of years of graduation are reported in Table 7. The distribution of this data is plotted in Figure 2.

*Table 7: Summary statistics for year of graduation.*

| Descriptives | Value |
|---|---|
| Count | 52 |
| Mean | 2015 |
| Std. deviation | 6.845637 |
| Minimum | 1991 |
| 25% | 2011 |
| 50% | 2018 |
| 75% | 2020 |
| Maximum | 2023 |

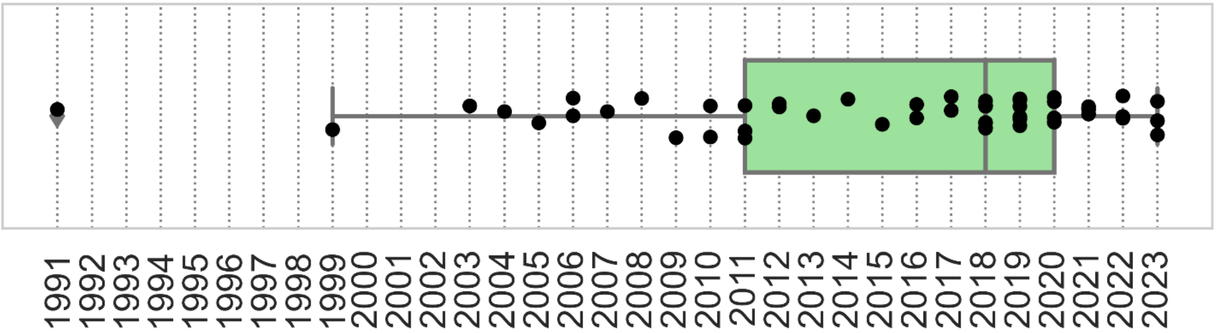


*Figure 2: Distribution of participants' years of graduation.*

## 1.4 Countries of origin, training, and residence

The summary of countries of origin, training, and residence are reported in tables 8 to 10. Table 11 contains counts for each combination of countries was produced.

*Table 8: Counts for country of origin.*

| Country of origin | Answers (n) | Answers (%) |
|---|---|---|
| Austria | 2 | 4% |
| Croatia | 1 | 2% |
| Denmark | 3 | 6% |
| France | 14 | 27% |
| Germany | 4 | 8% |
| Greece | 3 | 6% |
| Ireland | 1 | 2% |
| Italy | 4 | 8% |
| Norway | 1 | 2% |
| Poland | 4 | 8% |
| Portugal | 1 | 2% |
| Slovenia | 2 | 4% |
| Spain | 1 | 2% |
| Switzerland | 1 | 2% |
| The Netherlands | 2 | 4% |
| United Kingdom | 7 | 14% |
| Total | 51 | 100% |

*Table 9: Counts for country of training.*

| Country of training | Answers (n) | Answers (%) |
|---|---|---|
| Austria | 1 | 2% |
| Croatia, Austria | 1 | 2% |
| Cyprus | 1 | 2% |
| France | 13 | 30% |
| Germany | 2 | 5% |
| Germany, Spain | 1 | 2% |
| Greece | 1 | 2% |
| Ireland, United Kingdom | 1 | 2% |
| Italy | 2 | 5% |
| Poland | 4 | 9% |
| Portugal | 1 | 2% |
| Sweden | 1 | 2% |
| Switzerland | 1 | 2% |
| The Netherlands | 3 | 7% |
| United Kingdom | 10 | 23% |
| United Kingdom, Germany | 1 | 2% |
| Total | 44 | 100% |

*Table 10: Counts for country of residence.*

| Country of residence | Answers (n) | Answers (%) |
|---|---|---|
| Austria | 3 | 6% |
| Belgium | 3 | 6% |
| Croatia | 1 | 2% |
| Cyprus | 1 | 2% |
| Czech Republic | 1 | 2% |
| Denmark | 2 | 4% |
| France | 14 | 27% |
| Germany | 4 | 8% |
| Greece | 1 | 2% |
| Italy | 3 | 6% |
| Poland | 3 | 6% |
| Portugal | 1 | 2% |
| Slovenia | 2 | 4% |
| Spain | 1 | 2% |
| Switzerland | 1 | 2% |
| The Netherlands | 3 | 6% |
| United Kingdom | 8 | 15% |
| Total | 52 | 100% |

*Table 11: Table summarizing reported countries of origin, training, and residence.*

| Country of origin | Country of training | Country of residence | Count |
|---|---|---|---|
| Austria | Austria | Austria | 1 |
| Austria | United Kingdom, Germany | Austria | 1 |
| Croatia | United Kingdom | Croatia | 1 |
| Denmark | Sweden | Denmark | 1 |
| Denmark | The Netherlands | Belgium | 1 |
| France | France | Belgium | 1 |
| France | France | France | 12 |
| Germany | Germany | Germany | 2 |
| Germany | Germany, Spain | Germany | 1 |
| Greece | Cyprus | Cyprus | 1 |
| Greece | Greece | Greece | 1 |
| Ireland | Ireland, United Kingdom | United Kingdom | 1 |
| Italy | Italy | Italy | 1 |
| Italy | Italy | United Kingdom | 1 |
| Norway | United Kingdom | United Kingdom | 1 |
| Poland | Poland | Czech Republic | 1 |
| Poland | Poland | Poland | 3 |
| Portugal | Portugal | Portugal | 1 |
| Slovenia | United Kingdom | Slovenia | 1 |
| Switzerland | Switzerland | Switzerland | 1 |
| The Netherlands | The Netherlands | The Netherlands | 2 |
| United Kingdom | United Kingdom | The Netherlands | 1 |
| United Kingdom | United Kingdom | United Kingdom | 5 |

To infer whether the participants moved to receive training, the series of answers about countries of origin, training, and current residence were sorted into different categories (Figure 3). Most of the participants (n = 31, 67%) did not move to other countries, while six participants (13%) moved to another country for training and subsequently returned to their country of origin. The rest of the participants either moved for training and stayed there, moved at each step of the way (between the countries of origin and training, and between countries of training and current residence), or were trained in the country of origin and moved after that.

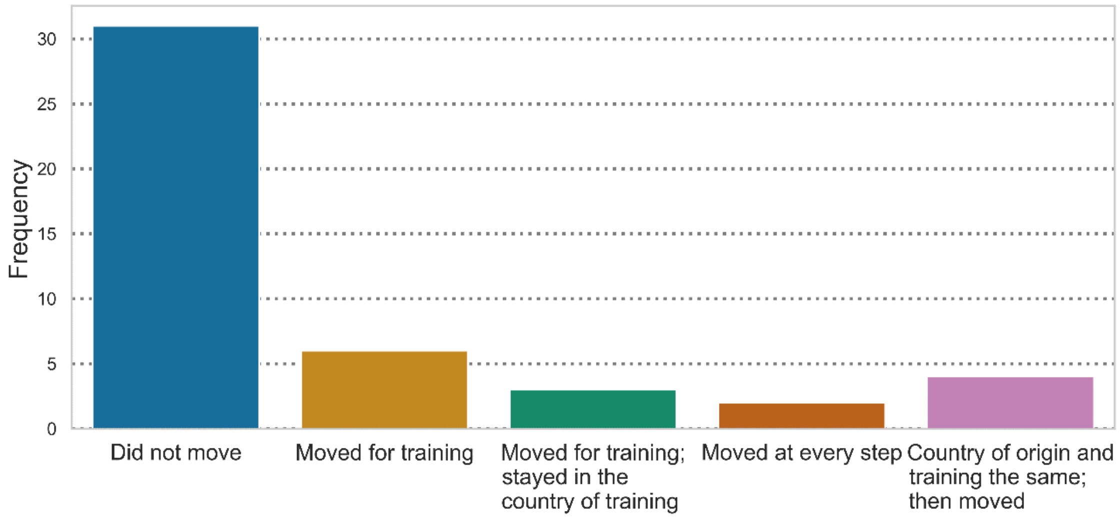


*Figure 3: Movements of the participants between countries of origin, training, and residence.*

# 2.Training and experience

Counts of answers for the questions regarding training that participants received are summarized in Table 12. Participants were also asked whether enough time was spent on the topic, and these results are summarized in Tables 13 and 14. Comments specifying the types of extracurricular training are reported in Table 15.

*Table 12: Table with counts for questions regarding training. Left: counts of participants that had training on cremation analysis included in their syllabus. Middle: counts of participants that received training in cremation outside their syllabus. Right: Counts of participants who received or did not receive any kind of training in cremation analysis before working with these remains.*

| | Syllabus | | | Extracurricular | | | Any training | |
|---|---|---|---|---|---|---|---|---|
| | Answers (n) | Answers (%) | | Answers (n) | Answers (%) | | Answers (n) | Answers (%) |
| **No** | 27 | 61% | | 23 | 59% | | 19 | 43% |
| **Yes** | 17 | 39% | | 16 | 41% | | 25 | 57% |
| **Total** | 44 | 100% | | 39 | 100% | | 44 | 100% |

*Table 13: Counts of 'yes' and 'no' answers to whether enough time was spent on cremation deposits in the syllabi.*

| Enough time spent on topic? | Answers (n) | Answers (%) |
|---|---|---|
| **No** | 18 | 67% |
| **Yes** | 9 | 33% |
| **Total** | 27 | 100% |

*Table 14: Summary of the comments about training on cremated remains as a part of syllabi.*

| Was enough time spent of the topic |
|---|
| More could have been taught, but it is also difficult to teach. |
| No memory of specific classes, but if there were, they were surely short and not exhaustive. |
| Only few courses in master's program in the common curriculum. |
| Cremation analysis requires a lot of experience, only basics can be conveyed through teaching. |
| Syllabus conveyed only basic theoretical knowledge. |
| Only three days of internship. |
| There was a general presentation on archaeological cremation. |
| The training was too general, especially considering that 35 people were attending the class. |

*Table 15: Summary of comments on extracurricular courses participants attended.*

| Types of extracurricular courses attended by participants |
|---|
| Private training by a cremation specialist in the context of a research dissertation. |
| A course and a seminar. |
| A six-month internship in the context of a master's degree. |
| Trained in cremation analysis as a part of experimental archaeology experiment. |
| Two weeks of training under guidance of a cremation specialist. |
| Trained in cremation analysis by master thesis supervisor. |
| Three weeks of intensive training at a major cremation site in identification of bones, data analysis, interpretation, and writing of anthropological reports. |
| Practical research course. |
| Seminar in funerary archaeology with different optional topics. |
| Summer school. |

Participants’ experience with the analysis was assessed via the number of cremation deposits analysed in participants’ careers. The descriptive statistics for these data, and the normality tests are listed in Tables 16 and 17.

*Table 16: Descriptive statistics for the number of cremation deposits analysed in participants' careers.*

| Cremations analysed in career | | Statistic | Std. Error |
|---|---|---|---|
| **Mean** | | 366.09 | 107.417 |
| **95% Confidence Interval for Mean** | **Lower Bound** | 149.32 | |
| | **Upper Bound** | 582.87 | |
| **5% trimmed mean** | | 243.72 | |
| **Median** | | 60.00 | |
| **Variance** | | 496150.134 | |
| **Std. Deviation** | | 704.379 | |
| **Minimum** | | 2 | |
| **Maximum** | | 3000 | |
| **Range** | | 2998 | |
| **Interquartile range** | | 270 | |
| **Skewness** | | 2.926 | .361 |
| **Kurtosis** | | 8.435 | .709 |

*Table 17: Normality tests for cremation deposits analysed in participant’s' careers.*

| Kolmogorov-Smirnov | | | Shapiro-Wilk | | |
|---|---|---|---|---|---|
| Statistic | df | Sig. | Statistic | df | Sig. |
| .305 | 43 | *<.001* | .543 | 43 | *<.001* |

# 3.Working environment

## 3.1 Number of experts analysing cremated deposits in participants' laboratories

Participants were asked how many people including them work on cremated deposits in their laboratories and institutions (Table 18), to see whether they are surrounded by experts with similar background).

*Table 18: Descriptive statistics for the number of people studying cremated remains in participants' laboratories and institutions.*

| How many people in your lab, including you, do osteology on cremations? | | Statistic | Std. Error |
|---|---|---|---|
| Mean | | 2.19 | .363 |
| 95% Confidence Interval for Mean | Lower Bound | 1.45 | |
| | Upper Bound | 2.93 | |
| 5% Trimmed Mean | | 1.88 | |
| Median | | 2.00 | |
| Variance | | 4.880 | |
| Std. Deviation | | 2.209 | |
| Minimum | | 0 | |
| Maximum | | 10 | |
| Range | | 10 | |
| Interquartile Range | | 1 | |
| Skewness | | 2.607 | .388 |
| Kurtosis | | 7.289 | .759 |

## 3.2 Other analyses performed by labs and participants

Respondents' degree of specialisation was assessed by looking into whether their laboratory (Table 19) or themselves (Table 20) perform other types of analyses than osteology on burnt bones.

*Table 19: Other analyses performed by laboratories and institutions were participants work.*

| Other analyses | Answers (n) | Answers (%) |
|---|---|---|
| None | 26 | 60% |
| Isotopes, C14 | 5 | 12% |
| Isotopes | 4 | 9% |
| Spectroscopy | 3 | 7% |
| Isotopes, Spectroscopy | 2 | 5% |
| Histology | 1 | 2% |
| C14 | 1 | 2% |
| C14, Palaeogenetics | 1 | 2% |
| Total | 43 | 100% |

*Table 20: Number of participants conducting analyses other than osteology on cremated remains.*

| Analyses other than osteological analyses performed by participants | Answers (n) | Answers (%) |
|---|---|---|
| No | 21 | 58% |
| Yes | 15 | 42% |
| Total | 36 | 100% |

## 3.3 Sector and current position

As cremated deposits are encountered for analysis in different sectors, the participants were asked which sector they work in (Table 21) and what professional position they occupy (Table 22). For further statistical tests concerning the 'sector' variable in the following sections, only academia, commercial and public sector were considered, due to a small number of datapoints for other sectors.

*Table 21: Counts of participants per sector.*

| Sector | Answers (n) | Answers (%) |
|---|---|---|
| Academia | 22 | 54% |
| Commercial | 8 | 20% |
| Heritage organisation | 2 | 5% |
| Museum | 2 | 5% |
| Public sector | 7 | 17% |
| Total | 41 | 100% |

*Table 22: Counts of participants in the different positions.*

| Current position | Answers (n) | Answers (%) |
|---|---|---|
| Commercial | 8 | 20% |
| Public sector | 7 | 17% |
| PhD student | 7 | 17% |
| Professor | 7 | 17% |
| Postdoc | 3 | 7% |
| Heritage organisation | 2 | 5% |
| Research assistant | 2 | 5% |
| Museum | 2 | 5% |
| PhD student, Research assistant | 2 | 5% |
| Bachelor | 1 | 2% |
| Total | 41 | 100% |

## 3.4 Language at work

The most frequently used language was English, with (n = 29, 73%) of participants using it at work either as the main or secondary language, followed by French (n = 14, 35%). German was used by 7 participants (18%), Italian and Polish by 4 (10%), Dutch by 3 (8%), Spanish by 2 (5%), and Czech, Danish, Greek, Portuguese, and Slovene by 1 (3%). The full summary of language combinations used at work is reported in Table 23.

*Table 23: Work language combinations used by participants.*

| Language 1 | Language 2 | Language 3 | Language 4 | count |
|---|---|---|---|---|
| Danish | | | | 1 |
| Dutch | | | | 1 |
| English | Dutch | French | | 1 |
| English | German | | | 2 |
| English | Italian | | | 1 |
| English | | | | 10 |
| English | Polish | Spanish | | 1 |
| French | Dutch | English | | 1 |
| French | English | German | Italian | 1 |
| French | English | Italian | | 1 |
| French | English | | | 3 |
| French | English | Spanish | | 1 |
| French | | | | 6 |
| German | English | | | 1 |
| German | | | | 2 |
| Greek | English | | | 1 |
| Italian | English | | | 1 |
| Polish | Czech | English | German | 1 |
| Polish | English | | | 1 |
| Polish | | | | 1 |
| Portuguese | English | | | 1 |
| Slovene | English | | | 1 |

## 3.5 Analysis of cremated remains part of current or past job

To verify whether the respondents were experts working with cremated remains, they were asked whether analysing cremation deposits was part of their current job (Table 24). To assess whether to further analyse the answers of the participants who do not currently work with cremated remains, their answers were matched against how many deposits they have studied in their careers. The results are reported in Table 25.

*Table 24: Counts of answers as to whether analysing cremated remains is a part of participants' current job.*

| Is osteological analysis of cremated remains part of your current job? | Answers (n) | Answers (%) |
|---|---|---|
| **No** | 6 | 14% |
| **Other** | 3 | 7% |
| **Yes** | 35 | 80% |
| **Total** | 44 | 100% |

*Table 25: Number of cremated deposits analysed by participants who do not currently work with cremated remains or who did not specify the answer to that question.*

| Cremation deposits analysed in career | Is the osteological analysis of cremated remains a part of your current job? |
|---|---|
| 10 | No |
| 10 | No |
| 20 | No |
| 20 | No |
| 40 | No |
| 300 | No |
| 2 | Other (not specified) |
| 40 | Other (not specified) |
| 100 | Other (not specified) |

## 3.6 Percent of working time spent on cremated deposits

To find out more about the amount of time that participants spend on analysing cremation deposits at work, they were asked to report the approximate percentage of working time they spend on this task. The descriptive statistics and normality distributions are reported in Tables 26 and 27.

*Table 26: Descriptive statistics for percentage of working time spent on cremated remains.*

| Percentage of working time spent on cremated remains | | Statistic | Std. Error |
|---|---|---|---|
| Mean | | 29.13 | 4.746 |
| 95% Confidence Interval for Mean | Lower Bound | 19.52 | |
| | Upper Bound | 38.75 | |
| 5% Trimmed Mean | | 27.08 | |
| Median | | 20.00 | |
| Variance | | 855.847 | |
| Std. Deviation | | 29.255 | |
| Minimum | | 0 | |
| Maximum | | 100 | |
| Range | | 100 | |
| Interquartile Range | | 40 | |
| Skewness | | 1.069 | .383 |
| Kurtosis | | -.176 | .750 |

*Table 27: Normality test results for percentage of working time spent on cremated remains.*

| Kolmogorov-Smirnov | | | Shapiro-Wilk | | |
|---|---|---|---|---|---|
| **Statistic** | df | Sig. | Statistic | df | Sig. |
| **.280** | 38 | *<.001* | .825 | 38 | *<.001* |

## 3.7 Excavation

Cremated remains can reach the stage of osteological analysis when already excavated and bagged, or the osteologists must excavate the deposits in the laboratory or in the field. Excavating the deposits or not, might also influence the analysis protocols and capturing of additional contextual information about the deposits. Participants were therefore asked to report the approximate percentage of studied deposits that they also excavated themselves. The descriptive statistics and normality tests for this data are reported in Tables 28 and 29.

*Table 28: Descriptive statistics for the percentage of studied deposits that osteologists excavate themselves.*

| **Percent of cremation deposit excavated** | | **Statistic** | **Std. Error** |
|---|---|---|---|
| **Mean** | | 38.89 | 6.150 |
| **95% Confidence Interval for Mean** | Lower Bound | 26.40 | |
| | Upper Bound | 51.37 | |
| **5% Trimmed Mean** | | 37.65 | |
| **Median** | | 30.00 | |
| **Variance** | | 1361.702 | |
| **Std. Deviation** | | 36.901 | |
| **Minimum** | | 0 | |
| **Maximum** | | 100 | |
| **Range** | | 100 | |
| **Interquartile Range** | | 76 | |
| **Skewness** | | .347 | .393 |
| **Kurtosis** | | -1.487 | .768 |

*Table 29: Normality tests for the percentage of studied deposits that osteologists excavate themselves.*

| **Kolmogorov-Smirnov** | | | **Shapiro-Wilk** | | |
|---|---|---|---|---|---|
| **Statistic** | df | Sig. | Statistic | df | Sig. |
| **.228** | 36 | *<.001* | .850 | 36 | *<.001* |

The difference between the percentage of excavated deposits were compared across sectors and across the first language indicated by respondents. While not ideal, first work language rather than country or residence was chosen to have more datapoints available per category (main file, Working conditions and environment section).

It should be noted that sector and language were found to be significantly related (see the Participants section of the main article).

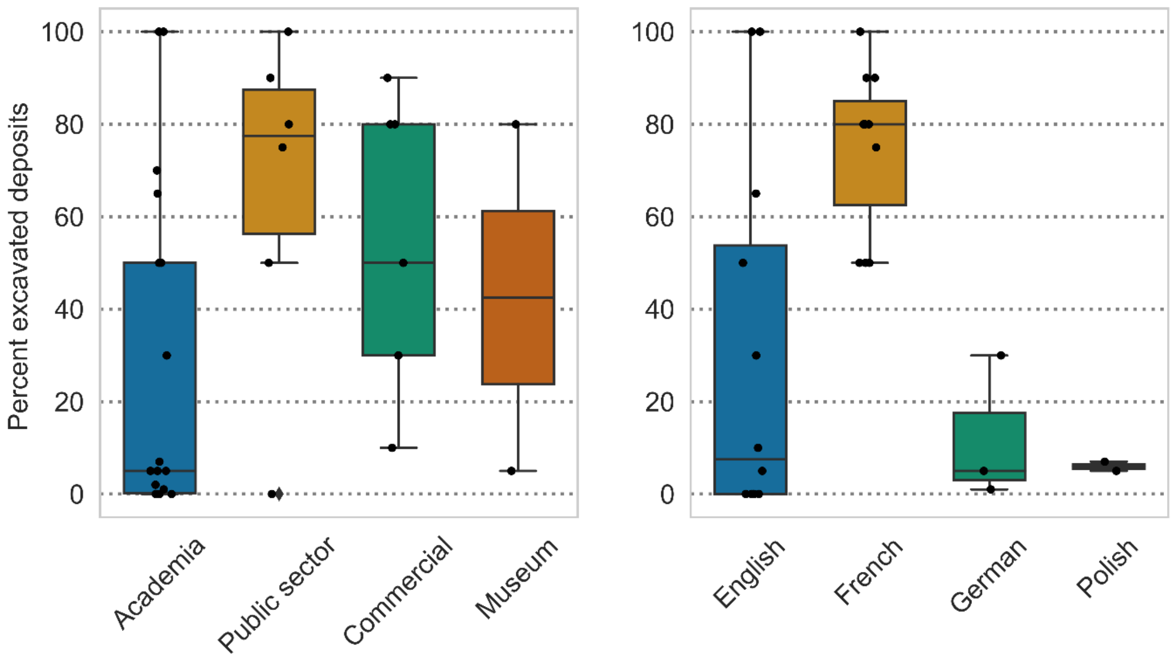


*Figure 4: Percentage of cremation deposits excavated by the participants per sector (left) and per 1st language (right) listed. Heritage organization is not included in the plot, because only one data point was available, and languages with only one data points were excluded.*

## 3.8 Pressure

To assess whether the data production of the analysis of cremation deposits influenced the experts working with burnt human remains, the participants were asked whether they felt any pressure to produce a clear sex estimation from cremated remains (Table 30). The relationship in pressure to produce clear sex estimation and gender was not significant ($\chi^2$ (1, $N$ = 36) = 0.03, $p$ = .958). The relationship between sectors in terms of feeling the pressure for a clear sex estimation from cremation deposits, although more pronounced than for gender, was also not significant ($\chi^2$ (2, $N$ = 34) = 5.057, $p$ = .080).

*Table 30: Counts of participants feeling the pressure to produce a clear sex estimation for cremation deposits.*

| Pressure clear estimation | Answers (n) | Answers (%) |
|---|---|---|
| No | 27 | 73% |
| Yes | 10 | 27% |
| Total | 37 | 100% |

Additionally, respondents were asked whether they felt pressured to analyse the cremation deposits and to produce reports about their analyses on a 5-point scale, where 1 was little pressure and 5 was a lot of pressure. Pressure to analyse and report on the cremation deposits was also not statistically significantly different across genders according to the Mann-Whitney U test results (analysis: $z$ = -0.899, $p$ = .368; report: $z$ = -0.368, $p$ = .171).

Kruskal-Wallis test was performed to establish potential differences in pressure for analysis and reporting on cremation deposits and sector (academia, public sector, commercial). The difference was not statistically significant for either (analysis vs. sector: $\chi^2$(2, $N$ = 31) = 2.676, $p$ =

.262; report vs. sector: $\chi^2(2, N = 31) = 3.800$, $p$ =.150). However, there was a slight difference in pressure to produce report between academia and public sector ($p$ = .061).

Mann-Whitney U tests were performed to see whether there was a difference in pressure between French-speaking and other participants, and it was not significant for analysis ($z$ = -0.848, $p$ = .065), and on the limit of significance for report ($z$ = -1.957, $p$ = .050).

The two pressure distributions (analysis vs. report) were compared using Wilcoxon Signed Ranks tests and were not statistically significantly different ($Z$ = -0.367, $p$ = .714).

# 4. Analysis protocols

## 4.1 Manuals and lab forms, descriptions of protocols

Different variables pertaining to the protocols that participants use in studying cremation deposits are reported here. The counts of labs where protocols are formalized via user manuals is summarized in Table 31, and those that use recording forms in Table 32.

*Table 31: Number of labs that have their own manuals for studying cremation deposits.*

| Is there a cremation analyses manual in your lab? | Answers (n) | Answers (%) |
|---|---|---|
| No | 22 | 65% |
| Yes | 12 | 35% |
| Total | 34 | 100% |

*Table 32: Number of labs that have their recording sheets specifically for cremation deposits.*

| Is there a cremation analyses form in your lab? | Answers (n) | Answers (%) |
|---|---|---|
| No | 17 | 53% |
| Yes | 15 | 47% |
| Total | 32 | 100% |

Whenever respondents indicated that no formalized manual and recording sheets were used in their laboratories, they were asked to describe their analysis. Two different examples of protocols described by participants are listed in Table 33.

*Table 33: Two different examples of protocol analysis provided by the participants. The text was restructured for better readability. Example 2 was translated from French. The spreadsheet that example 2 is referring to is designed to calculate different weight indices and this assess the completeness of different skeletal regions.*

| EXAMPLE 1 | EXAMPLE 2 |
|---|---|
| • Sieving: fraction sizes >10mm, 5-10 mm, 2-5mm, <2mm.<br>• Assessment of fractures and burning stage.<br>• Separating human bone from animal bone, artifacts, and charcoal.<br>• Sorting bones based on skeletal regions: skull, torso, upper limbs, lower limbs, unidentified diaphyses, epiphyses, carpals/tarsals, and rest.<br>• Recording and siding all identifiable bones and anatomical features.<br>• Estimation of MNI.<br>• Estimation of age-at-death (non-adults: tooth development, epiphyseal fusion; adults: cranial sutures, transitional analysis; all: tooth cementum annulation analysis, histological age assessment).<br>• Morphological and metric sex estimation using cranial and pelvic morphological features, and metrics after Cavazutti et al. (2019). | Protocol for excavating an urn depends on bone quantity, preservation of the remains, type of sediment (if present), and available time.<br>• Excavation by layers: every bone is isolated, identified, numbered, photographed, cleaned, and conditioned.<br>• Matching fragments of the same bones together when possible.<br>• Estimation of MNI: sorting by anatomical region, identifying duplicate skeletal elements, morphological incompatibilities, and differences in age-at-death.<br>• Separating animal bones and artifacts.<br>• Weighing each bone by anatomical region.<br>• Entering data into a spreadsheet designed by Depierre and Duday (the last version is from 2014).<br>• This is the consensus method for studying cremations in France. |

## 4.2 Elements of cremation analysis and priorities when under time constraints

Different parameters can be assessed when analysing cremation deposits. Table x in the main article lists these parameters and how many of the participants analyse them. Table 34 lists which of the features participants consider priority for assessment when they are under time constraint.

*Table 34: Order of priorities in which participants assess cremated deposits when under time constraints.*

| Rank 1 | Rank 2 | Rank3 | Rank 4 | Rank 5 | Rank 6 | Rank 7 |
|---|---|---|---|---|---|---|
| Age | Sex | MNI | Burning degree | Maximum fragment size | Weights of skeletal categories | Weights size fractions |
| Burning degree | Weights size fractions | Maximum fragment size | MNI | Age | Sex | Weights of skeletal categories |
| Maximum fragment size | Burning degree | | | | | |
| MNI | Age | Weights of skeletal categories | Burning degree | Maximum fragment size | Weights size fractions | Sex |
| MNI | Burning degree | Sex | Weights of skeletal categories | Maximum fragment size | Weights size fractions | Age |
| MNI | Weights of skeletal categories | Burning degree | Maximum fragment size | Sex | Age | Weights size fractions |
| Weights size fractions | MNI | Maximum fragment size | Burning degree | Age | Sex | Weights of skeletal categories |

# 5.Sex estimation

## 5.1 Most reliable methods for sex estimation

Answers pertaining to the trust in different methods are presented in Table 35. Open comments to the question on which methods they trust most are listed in Table 36.

*Table 35: Methods most trusted by the participants.*

| Type of methods | Answers (n) | Answers %) |
|---|---|---|
| **Morphological** | 22 | 71% |
| **Morphological and metric** | 3 | 10% |
| **Metric** | 2 | 6% |
| **Other (not specified)** | 2 | 6% |
| **None** | 2 | 6% |
| **Total** | 31 | 100% |

*Table 36: Comments on reliability of the methods.*

| Most reliable methods for sex estimation - comments |
|---|
| As the fragmentation level of the deposits, I'm analysing is usually high I found it very unlikely to find even minimal number of the fragments needed for multivariate method. I don't believe any metric assessment that is not multivariate method is a reliable one. |
| None. |
| None, given the deformation of bone under the effect of heat and its high fragmentation. |
| Combination of morphological and metric methods. |
| Given the state of preservation, a metric approach is rarely possible in the deposits I study. |
| Dependent on sample size and bone condition: morphological assessment needs seriation of frequently observed elements for population calibration; metric needs individual probabilities being a male or a female. |
| Due to the lack of other credible methods, I am using the traditional methods of paleodemo data based on morphology. It is biased, especially if we consider level of shrinkage/distortion and devote our observations specifically to the specific populations. |
| It really depends on the preservation state of the remains. |
| Metric assessments are not useful in cremated bone as thermal changes to bone structure do not allow for accurate standard measurements. Measurements on cremated bone can be difficult to replicate given individual changes to thermal bone that can differ based on temperature, body position, pyre, etc. |
| Multiple methods. |
| Of course, a combination of both metric and morphological would be my preference. |
| The morphological methods can almost never be applied due to incomplete pelvic bones and deformation and fragmentation of the bones by fire. |

## 5.2 Methods used by participants

Methods used by the participants are presented in Table 4 of the main article.

The potential difference between the methods used by the participants and the area of expertise were assessed via a Chi-Square test.

Chi-Square tests was performed to evaluate the relationship between the degree fields, areas of expertise, sector, and the 1st working language to the methods used (Table 37).

There were no significant relationships between the types of methods used by the participants and the degree field they specified. There was a statistically significant relationship between the area of expertise and the use of morphological methods, where the only participants not to use it selected anthropology as main area of expertise. Participants from the public sector used less morphological methods (the same participants indicated they do not sex cremations, because methods are not reliable), and they also less often use overall size and robusticity as sex indicator. A similar pattern was shown for language, where there is a tendency for French-speaking participants to use less morphological methods, but also almost none use the metric methods. The same observation is true for the overall size and robusticity.

The last two comparisons are also similar due to the significant relationship between French-speaking participants working in the public sector (see Language at work section).

*Table 37: Relationship between degree fields, main area of expertise, sector, 1st language at work and methods used.*

| METHOD TYPE | Degree field (df=1, n=33) | | Area of expertise (df=5, n=31) | | Sector (df=2, n=29) | | Language (df=2, n=24) | |
|---|---|---|---|---|---|---|---|---|
| | χ2 | p-value | χ2 | p-value | χ2 | p-value | χ2 | p-value |
| **Morphological** | 0.214 | .643 | 11.117 | *.049** | 6.751 | *.034** | 5.714 | .057 |
| **Metric** | 0.247 | .619 | 7.196 | .206 | 5.591 | .061 | 6.993 | *.030** |
| **Lateral angle** | 0.928 | .335 | 1.748 | .883 | 1.940 | .379 | 2.667 | .264 |
| **Bony labyrinth** | 1.384 | .239 | 2.359 | .798 | 2.611 | .271 | 2.182 | .336 |
| **Overall size and robusticity** | 0.047 | .829 | 8.119 | .150 | 11.869 | *.003** | 9.778 | *.008** |
| **Unpublished methods** | 3.275 | .070 | 7.330 | .197 | 0.633 | .729 | 7.304 | *.026** |
| **Unpublished metric references** | 0.051 | .822 | 8.119 | .150 | 0.590 | .744 | 3.636 | .162 |

## 5.3 Which areas of skeleton most reliable

Participants were asked to rank different skeletal regions from most to least reliable for sex estimation (Table 38).

*Table 38: Rankings of different skeletal areas from the most reliable to the least reliable.*

| Skeletal area | Rank 1 | Rank 2 | Rank 3 | Rank 4 | Rank 5 |
|---|---|---|---|---|---|
| **Pelvis** | 26 | 0 | 0 | 0 | 0 |
| **Skull** | 1 | 21 | 3 | 1 | 0 |
| **Hands / feet** | 0 | 2 | 14 | 13 | 19 |
| **Long bones** | 0 | 3 | 9 | 9 | 0 |
| **Other (not specified)** | 0 | 0 | 0 | 2 | 7 |

## 5.4 Most often found sexually dimorphic features

To be able to see how compatible the trust in different methods was with what elements were most often found in cremated remains people study, they were asked to rank different

morphological (Table 39) and metric features (Table 40) from those that they found most often to least often.

*Table 39: Elements for morphological sex assessment most often found in cremated deposits by the participants.*

| Skeletal element | Rank 1 | Rank 2 | Rank 3 | Rank 4 | Rank 5 | Rank 6 | Rank 7 | Rank 8 | Rank 9 | Rank 10 | Rank 11 |
|---|---|---|---|---|---|---|---|---|---|---|---|
| **Sciatic notch** | 10 | 1 | 0 | 0 | 4 | 4 | 0 | 2 | 1 | 0 | 1 |
| **Mastoid process** | 7 | 6 | 4 | 5 | 0 | 2 | 0 | 0 | 0 | 0 | 0 |
| **Supraorbital margin** | 6 | 3 | 2 | 3 | 2 | 0 | 0 | 7 | 1 | 0 | 0 |
| **Nuchal crest/Occipital protuberance** | 2 | 6 | 9 | 2 | 4 | 3 | 2 | 0 | 0 | 0 | 0 |
| **Supraorbital ridge** | 2 | 3 | 5 | 4 | 3 | 4 | 1 | 1 | 1 | 0 | 0 |
| **Posterior zygomatic arch** | 1 | 1 | 1 | 1 | 1 | 4 | 5 | 3 | 2 | 1 | 1 |
| **Pubic bone length** | 1 | 2 | 1 | 1 | 1 | 2 | 1 | 0 | 1 | 2 | 0 |
| **Subpubic angle** | 1 | 1 | 3 | 1 | 0 | 0 | 1 | 3 | 1 | 0 | 0 |
| **Gonial angle** | 0 | 1 | 2 | 2 | 3 | 0 | 4 | 0 | 0 | 0 | 1 |
| **Mental eminence** | 0 | 0 | 2 | 4 | 3 | 1 | 2 | 2 | 1 | 1 | 1 |
| **Ventral arc** | 0 | 6 | 1 | 0 | 0 | 0 | 4 | 0 | 0 | 1 | 0 |

*Table 40: Elements for metric sex assessment most often found in cremated deposits by the participants.*

| Skeletal element | Rank 1 | Rank 2 | Rank 3 | Rank 4 | Rank 5 | Rank 6 | Rank 7 | Rank 8 | Rank 9 | Rank 10 | Rank 11 | Rank 12 | Rank 13 | Rank 14 | Rank 15 | Rank 16 |
|---|---|---|---|---|---|---|---|---|---|---|---|---|---|---|---|---|
| **Mandible** | 12 | 3 | 0 | 0 | 2 | 1 | 0 | 1 | 0 | 0 | 1 | 0 | 0 | 0 | 0 | 0 |
| **Axis (dens)** | 4 | 5 | 4 | 2 | 1 | 1 | 2 | 1 | 0 | 1 | 0 | 0 | 0 | 0 | 1 | 0 |
| **Radius (proximal)** | 4 | 3 | 4 | 3 | 0 | 1 | 2 | 2 | 1 | 0 | 0 | 0 | 0 | 0 | 0 | 0 |
| **Femur (head)** | 3 | 5 | 4 | 3 | 3 | 0 | 1 | 1 | 2 | 0 | 0 | 0 | 0 | 0 | 1 | 0 |
| **Humerus (distal)** | 2 | 3 | 2 | 1 | 2 | 4 | 1 | 0 | 1 | 0 | 0 | 1 | 0 | 0 | 1 | 0 |
| **Humerus (proximal)** | 1 | 2 | 3 | 4 | 3 | 1 | 0 | 2 | 0 | 1 | 0 | 0 | 0 | 1 | 0 | 0 |
| **Scapula (glenoid fossa)** | 1 | 2 | 1 | 1 | 2 | 2 | 2 | 0 | 0 | 0 | 0 | 2 | 0 | 2 | 0 | 0 |
| **First metatarsal** | 0 | 0 | 1 | 1 | 1 | 2 | 4 | 1 | 1 | 1 | 0 | 1 | 1 | 0 | 2 | 0 |
| **Hamate** | 0 | 0 | 0 | 0 | 1 | 1 | 0 | 0 | 0 | 2 | 2 | 1 | 3 | 2 | 1 | 0 |
| **Lunate** | 0 | 0 | 3 | 2 | 0 | 2 | 0 | 0 | 1 | 1 | 2 | 1 | 2 | 2 | 0 | 0 |
| **Navicular** | 0 | 0 | 0 | 0 | 0 | 0 | 1 | 2 | 2 | 6 | 4 | 0 | 2 | 3 | 1 | 0 |
| **Other** | 0 | 0 | 0 | 0 | 0 | 0 | 0 | 0 | 0 | 0 | 0 | 0 | 0 | 0 | 0 | 4 |
| **Patella** | 0 | 1 | 3 | 3 | 2 | 1 | 0 | 1 | 1 | 0 | 2 | 1 | 1 | 0 | 0 | 0 |
| **Scaphoid** | 0 | 0 | 0 | 2 | 2 | 1 | 0 | 0 | 2 | 1 | 0 | 1 | 2 | 1 | 2 | 0 |
| **Talus** | 0 | 1 | 0 | 0 | 2 | 0 | 3 | 3 | 2 | 0 | 1 | 3 | 0 | 0 | 0 | 0 |

## 5.5 Number of elements needed for sex estimation (lab and personal rule)

Respondents were asked how many elements their labs require to assign different sex estimations to the remains they study, and who many they require themselves (Fig 3 in the main text). Although the participants seem to require slightly more elements than what is required by the labs, the difference is not significant (Table 41).

*Table 41: Statistical differences between required fragments for sex estimation between labs and participants.*

| | Statistic | p-value |
|---|---|---|
| **Ambiguous** | 13 | 0.865 |
| **Possible female/male** | 16,5 | 0.472 |
| **Probable female/male** | 14,5 | 0.621 |
| **Female/male** | 15 | 0.670 |
| **Indeterminate** | 26 | 0.877 |

## 5.6 Checking own estimates, checking with colleagues and platforms

To see whether participants were checking their results with other colleagues or by themselves, they were asked how frequently this happens (Table 42). They were also asked whether they were using platforms to ask questions (Table 43).

*Table 42: Frequency with which participants double check their cremated deposits.*

| Frequency | Checking own results a second time | Results checked by colleagues |
|---|---|---|
| **Always** | 3 | 6 |
| **Often** | 7 | 8 |
| **Sometimes** | 16 | 10 |
| **Never** | 5 | 1 |

Platforms were used sporadically by the participants (Table 43).

*Table 43: The use of online platforms and social media for help with sexing cremation deposits.*

| Do you use online platforms (e.g. JiscMail, Facebook Groups, Twitter) to discuss and aid in the identification of cremated remains? If so how frequently do you use these platforms? | |
|---|---|
| **No** | 14 |
| **Yes** | 3 |
| **Rarely** | 2 |
| **Comments** | I sometimes help with identification on FB group. |
| | We are not able to discuss commercial finds on social media. |
| | We have a group on Gmail. We all use it very frequently. |
| | Facebook Groups and Twitter. |
| | Did it once. |

## 5.7 Comfort

Participants were asked how comfortable they felt to assign sex estimates to cremation deposits, and how comfortable they felt trusting estimations from other colleagues on a scale from 1 to 10, where 1 was extremely comfortable, and 10 was extremely uncomfortable (Figure 5).

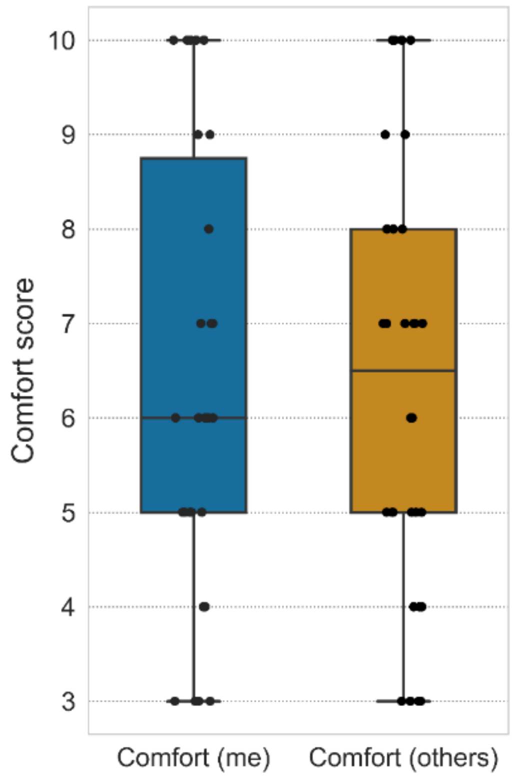


*Figure 5: Boxplots showing the answers on how comfortable participants felt estimating sex of cremation deposits and how comfortable they felt trusting other experts' estimates. The scale was from 1 (extremely comfortable) to 10 (extremely uncomfortable).*

Distributions of scores were also compared across genders (Figure 6). Mann-Whitney U tests were performed to test whether there was a difference between how comfortable each gender was with their own and others' estimates. No statistically significant differences were found for either case (own estimates between genders: $z = -1.068$, $p = .311$ (exact sig.); others' estimates between genders: $z = -1.612$, $p = .118$ (exact sig)).

The difference was also assessed within genders to see whether one gender group was more comfortable with their own or others' estimates. There was no statistically significant difference for either gender, based on the Wilcoxon signed ranks test that was performed on this data (Females: $Z = -0.058$, $p = .954$; Males: $Z = -0.552$, $p = .581$).

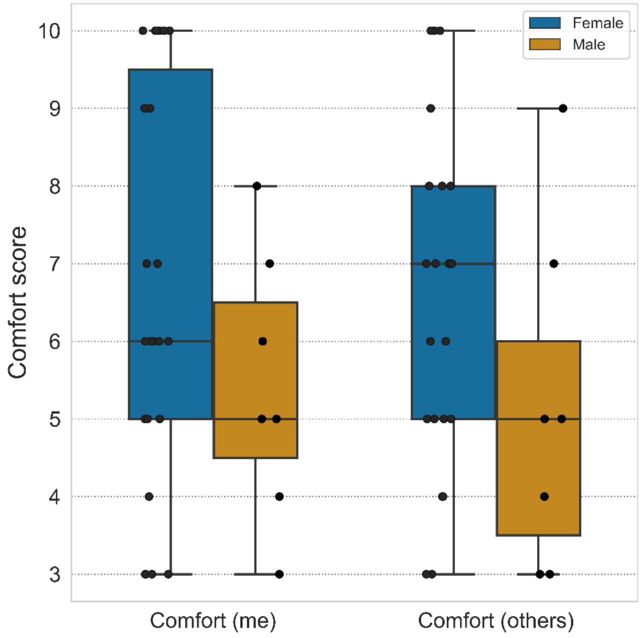


*Figure 6: Scores for comfort with own and others' sex estimates based on gender. The scale was from 1 (extremely comfortable) to 10 (extremely uncomfortable).*

To assess whether there was a correlation between comfort with own estimates and experience, Spearman's rho was calculated. There was a weak correlation between comfort and number of cremation deposits analysed in participants' careers $r_s(29) = -0.217$, $p = .241$), which was not statistically significant.

The comfort data for own estimates was also plotted against Sector (Figure 7) and Language (Figure 8), but no statistical analyses were performed due to small number of data points in each group.

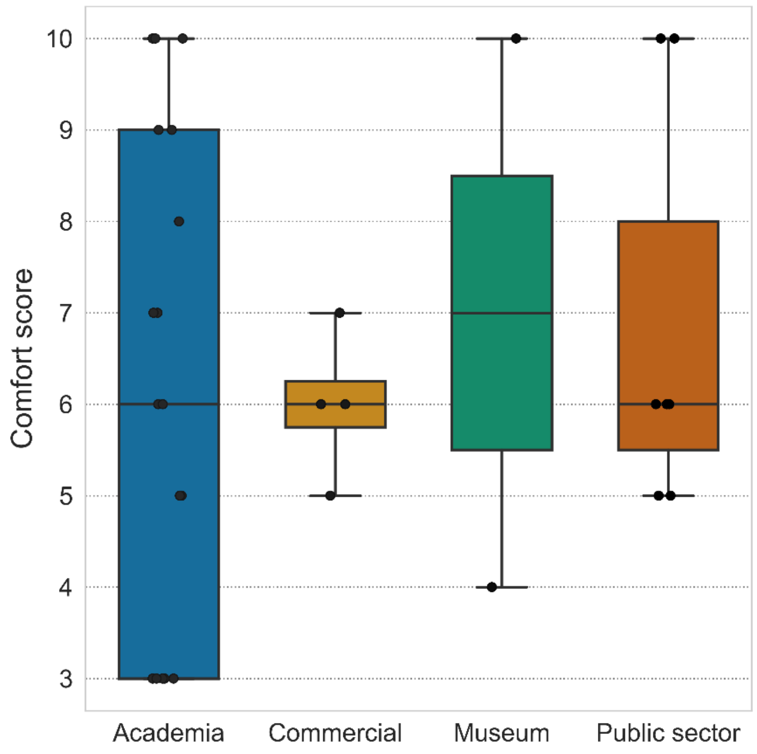


*Figure 7: Data scores for comfort with own estimates plotted against sector. The scale was from 1 (extremely comfortable) to 10 (extremely uncomfortable).*

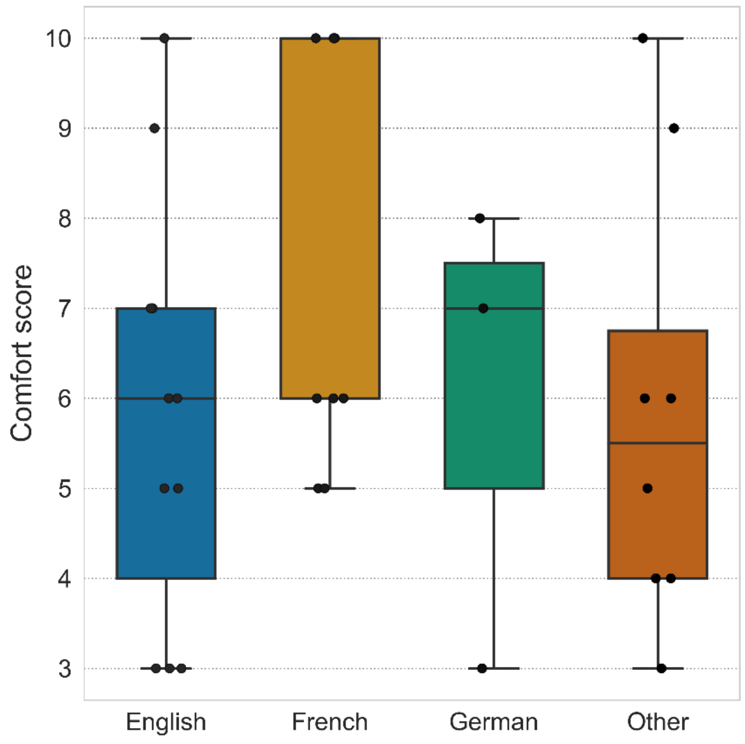


*Figure 8: Data scores for comfort with own estimates plotted against sector. The scale was from 1 (extremely comfortable) to 10 (extremely uncomfortable).*

# 6.Reporting

## 6.1 Publication of cremation analysis results

The types of publications in which participants publish their data is summarised in Table 5 in the main text.

## 6.2 Reporting sex in publications and reports

Reporting different methods and features can be different among the cremation experts. The way in which participants report on features and results of sexing in publications and reports is summarized in Table 44, Table 6 in the main text, and Fig 4 in the main text.

*Table 44: Elements of information about the dimorphic features participants include in their publications and reports.*

| Reporting on features | Always | Often | Sometimes | Never |
|---|---|---|---|---|
| **Listing all the dimorphic features found in the deposit** | 21 | 3 | 1 | 5 |
| **Listing the sex estimation for each of the reported features** | 19 | 3 | 2 | 5 |
| **Photographs of each dimorphic element** | 5 | 1 | 12 | 9 |
| **Expressing the degree of confidence about each feature** | 12 | 4 | 6 | 6 |
| **Expressing the degree of confidence for each individual** | 16 | 7 | 3 | 3 |
